# Metabolomic Analysis of Three Mollicute Species

**DOI:** 10.1371/journal.pone.0089312

**Published:** 2014-03-04

**Authors:** Anna A. Vanyushkina, Gleb Y. Fisunov, Alexey Y. Gorbachev, Dmitri E. Kamashev, Vadim M. Govorun

**Affiliations:** 1 Russian Institute of Physico-Chemical Medicine, Moscow, Russian Federation; 2 Russian Research Center Kurchatov Institute, Moscow, Russian Federation; 3 Shemyakin-Ovchinnikov Institute of Bioorganic Chemistry, Russian Academy of Sciences, Moscow, Russian Federation; 4 Moscow Institute of Physics and Technology (State University), Dolgoprudny, Moscow Region, Russian Federation; The University of Melbourne, Australia

## Abstract

We present a systematic study of three bacterial species that belong to the class Mollicutes, the smallest and simplest bacteria, *Spiroplasma melliferum*, *Mycoplasma gallisepticum*, and *Acholeplasma laidlawii*. To understand the difference in the basic principles of metabolism regulation and adaptation to environmental conditions in the three species, we analyzed the metabolome of these bacteria. Metabolic pathways were reconstructed using the proteogenomic annotation data provided by our lab. The results of metabolome, proteome and genome profiling suggest a fundamental difference in the adaptation of the three closely related Mollicute species to stress conditions. As the transaldolase is not annotated in Mollicutes, we propose variants of the pentose phosphate pathway catalyzed by annotated enzymes for three species. For metabolite detection we employed high performance liquid chromatography coupled with mass spectrometry. We used liquid chromatography method - hydrophilic interaction chromatography with silica column - as it effectively separates highly polar cellular metabolites prior to their detection by mass spectrometer.

## Introduction

In contrast to the abundance of systems-oriented approaches describing changes on the transcriptome or proteome level, relatively few studies examined the metabolome. Metabolomics studies are necessary to shed light on the link between genotypes and phenotypes. The goal of the presented research is to identify as many intracellular metabolites as possible in bacteria belonging to three species, namely *Spiroplasma melliferum*, *Mycoplasma gallisepticum*, and *Acholeplasma laidlawii*. All these bacteria are members of the class Mollicute, a unique category of bacteria that share the absence of a cell wall, a reduced genome, and simplified metabolic pathways. Mollicutes are widespread organisms in nature engaged in a parasitic lifestyle.

The poultry pathogen *M. gallisepticum* is a small bacterium that causes chronic respiratory disease in chickens and turkeys. *S. melliferum* is an insect pathogen with the capability to infect the honey bee [1]. *A. laidlawii* is a common contaminant of cell culture and has a broad variety of hosts, including bovines, the human oral cavity, birds and plants; it was originally isolated from sewage [2], [3], [4], [5], [6].

The mycoplasmic metabolome is significantly reduced, possibly due to its parasitic nature. Like all Mycoplasma, the three bacterial species lack some general metabolic pathways, including the TCA, amino acids synthesis, as well as purine and pyrimidine synthesis [7]. Genome-reduced organisms have limited adaptability to external factors [8]. We believe that comparison of the metabolomes of the three Mollicutes may reveal the primary adaptive features of the simplest metabolomes.

Metabolite identification in our study was performed by flow injection time-of-flight mass spectrometry (Q-TOF). Metabolite identity was confirmed by fragmentation of previously detected ions using targeted mass spectrometry. The liquid chromatography approach applied here, hydrophilic interaction chromatography (HILIC) with silica columns, effectively separates highly polar cellular metabolites for their subsequent detection using a high accuracy mass spectrometer in positive and negative acquisition modes [9].

Here we present reliable measurements of a hundred metabolites, including components of sugar, amino acid, and nucleotide metabolisms. We have identified about a third of all possible intracellular metabolites predicted by genome annotation and compared the metabolite content of three Mollicute species. Metabolomic data analysis is based on a previous proteogenomic study performed in our lab for all three bacterial species [3], [10], [11], [12]. The high-throughput-omics analysis, including transcriptomics, proteomics, and complemented with the metabolomic study performed in this work, will improve our comprehensive understanding how the three closely-related Mollicute species are regulated under different conditions [13].

## Materials and Methods

### Bacteria strains and growth conditions

*Spiroplasma melliferum* KC3 and *Acholeplasma laidlawii PG-8A* were kindly provided by Prof. G. Wroblewsky of the University of Rennes, France. The *Mycoplasma gallisepticum S6* strain was provided by Prof. S.N. Borkhsenius, Institute of Cytology,St. Petersburg, Russian Academy of Science. *S. melliferum* was grown in SP4 medum without any specific aeration and with mixing at 30°C for 20 hours after 1∶10 dilution, as described earlier [14]. *Acholeplasma laidlawii PG-8A* and *Mycoplasma gallisepticum S6* were grown in a modified Edward's medium (Tryptose 20 g/L, NaCl 5 g/l, NaOAc 5 g/l, KCl 1.3 g/l, Tris 3 g/l, yeast dialysate 5%, horse serum 6%, glucose 0.5%, pH 7.6) at 37°C for 20 hours after 1∶100 dilution [15]. Bacteria were cultured in covered flasks, and the time of growth and the pH of the solution were controlled [11]. Bacteria were harvested by centrifugation (10 min, 16,000 *g*) in the log phase, which was determined by pH values (6.5–6.6 for both, *S. melliferum* and *M. gallisepticum*). The metabolic profiles were obtained from cultures grown from a single clone for less than five passages. Each metabolome experiment was independently repeated at least seven times; 50 ml cultures from the same strain and with the same sample preparation procedure were used for each experiment.

### Determination of pH tolerance

We used the “color test” method to determine whether Mollicutes survive in acidic conditions and to estimate the critical pH value for each species. Aliquots of *M. gallisepticum*, *A. laidlawii* and *S. melliferum* culture were exposed to decreased pH (hydrochloric acid was added) in steps of ΔpH = 0.1 for 1 hour starting at pH 7.5. Each aliquot was then subjected to a series of dilutions (up to 10^9^) in medium (pH 7.5) containing pH indicator (Phenol Red), and the presence of living cells was detected by the medium color change. The tolerated acidity was defined as the minimum acidity at which 10% of cells survived.

### Reagents

The following chemicals were used as standards: sodium pyruvate (100 mg/ml, disodium salt hydrate), D-fructose 6-phosphate, hydrate of sodium phospho(enol)pyruvate (97% purity, enzyme quality), dehydrated disodium salt of D-ribose 5-phosphate, di-glyceraldehyde 3-phosphate (46.1 mg/ml). Purified (98%) amino acids, nucleotides, nucleosides (adenosine, deoxyadenosine, inosine, cytosine monophosphate, and thymidine) from Sigma-Aldrich (USA) were used as standards as well. The following reagents were used for extraction and solution preparation: absolute methanol (HPLC grade) from Biosolve (The Netherlands), ammonium acetate (ultra clean grade) from Helicon (Russia), formic acid (98–100%) from Riedel-de Haen (Germany), ammonium hydroxide solution (29.73%) from Fisher Scientific (USA), water (HPLC-MS) and acetonitrile (HPLC-MS) from Panreac (Spain).

### Extraction of metabolites

For metabolite analysis, *S. melliferum*, *M. gallisepticum*, or *A. laidlawii* (50 ml) were grown in a liquid medium. Cells were harvested by centrifugation at 16,000 g at 20°C for 10 min and washed with the washing solution (150 mM NaCl/50 mM Tris). The pH of the washing solution was 6.5 (the acidity in the logarithmic phase of Mycoplasma growth), while for acid stress studies the pH of washing solution was about 5.5 (depending on the type of culture and the selected conditions of acid stress). Thus, the culture was under the influence of selected acidity conditions during the washing procedure. The cell pellet was resuspended in 3 ml of washing solution (the pellet volume was 30 µl) and precipitated again. Then cells were washed twice with 150 µl of washing solution.

The metabolism was rapidly quenched by cold methanol extraction. A cold methanol extraction method was developed on the basis of a previously reported cold methanol extraction protocol [16], [17] as described below. The metabolites were extracted by adding 1000 µl of methanol (−77°C) to 75 ul of the resuspended cell pellet; the sample was vigorously shaken (1 min) and then kept at −77°C for 15 min. The sample was warmed for 3 min and then thoroughly shaken again. The resulting sample was centrifuged for 30 min at 16,000 g at 4°C. The supernatant was separated into aliquots and lyophilized. The dry extract was kept no longer than 4 days prior to analysis. The dry extract was dissolved in a mixture consisting of 20% acetonitrile and 80% water and analyzed immediately.

Until recently there was no published record of an LC-MS method of metabolome analysis of the studied bacteria (*S. melliferum*, *M. gallisepticum*, and *A. laidlawii*). Pre-testing a few other options (such as extraction by acetonitrile, methanol/water, acetonitrile/water/acid and others), we have chosen the cold methanol extraction method, as it has been demonstrated to be the most effective. The cold methanol extraction protocol described in [16] is the basis of the protocol used here. A similar modification has been successfully used recently for *M. pneumoniae* studies [18].

### Apparatus and HPLC/MS method

Mass spectrometry analysis was performed on a Q-TOF 6520 series time-of-flight mass spectrometer (Agilent Technologies). The flow from the analytical column was introduced directly into the electrospray ion source of the mass spectrometer. Prior to the experiment, the mass spectrometer was calibrated to 2 ppm accuracy of the *m/z* value. The ionizing spray voltage was 3500 V in both positive and negative ionization modes. Nitrogen of various degrees of purity was used as a dry gas at a pressure of 20 psi with a flow rate of 6 l/min and as a gas in the collision cell. The temperature of the quartz capillary was 325°C. HPLC-MS and HPLC-MS/MS analyses were carried out using the series 1200 high performance liquid chromatograph (Agilent Technologies), coupled with the mass spectrometer. The following chromatographic analytical column was used in the study: Zorbax RX-SIL Narrow-Bore (150 mm×2.1 mm×5 µm) from Agilent Technologies. To protect the separating phase of these highly effective analytical columns from chemical damage, as a safety cartridge we used the Zorbax RX-SIL 4-Pack analytical guard column (4.6 mm×12.5 mm×5 µm) purchased from Agilent Technologies.

Chromatographic analysis was performed with the following parameters: auto-sampling temperature, 8°C; analytical column temperature, 18°C; injection volume, 2 µl; solvent flow rate, 50 µl/min. The following solvents were used as eluting solutions: eluent A was 20 mM ammonium acetate/0.25 mM ammonium hydroxide in water/acetonitrile mixture of 95∶5 ratio, pH 8.02; eluent B was pure acetonitrile. The gradient of the solvent transition was as follows: for positive ionization mode *t* = 0, 100% B; *t* = 30 min, 0% B; *t* = 32 min, 0% B; *t* = 35 min, 100% B; *t* = 60 min, 100% B. In negative ionization mode *t* = 0, 100% B; *t* = 30 min, 0% B; *t* = 32 min, 0% B; *t* = 35 min, 100% B; *t* = 60 min, 100% B. Metabolite identification was confirmed by fragmentation spectra of the detected ions. The collision energy was fixed at 20 eV, and MS/MS spectra were recorded in the range of 30–1000 *m/z* with the minimal width of the ion isolation window at 1.3 *m/z*.

mzXML spectra results are available at: https://dl.dropboxusercontent.com/u/73633783/Spectra.rar

### Identification of metabolites and data processing software

Metabolite search and data processing were performed using the Metabolomic Analysis and Visualization Engine (MAVEN) [19] software and the Trans-Proteomic Pipeline (TPP) resource available online, where the data were converted into the mzXML format (MAVEN compatible). *S. melliferum*, *M. gallisepticum*, and *A. laidlawii* metabolites were identified using the list of all possible metabolites for these bacteria, garnered from all the protein annotation data that were previously reported [3], [11]. A list of all theoretically possible metabolites of *S. melliferum*, *M. gallisepticum*, and *A. laidlawii* was prepared in accordance with the KEGG database [20] and contained all the metabolites associated with the proteins that were annotated for these bacteria. The following parameters were used for the search: range of *m/z* values (extraction window), 15 ppm *m/z*; minimum intensity of peak, 1000 a.u.; minimum value of baseline signal intensity ratio, 10. If an identified metabolite was detected in at least five MS runs out of seven in specie one, while in specie two it was not detected, or it was detected only once, we attributed this metabolite to be present in the first species and to be absent in the second one.

### Comparative analysis of the *S. melliferum*, *M. gallisepticum*, and *A. laidlawii* metabolomes

A pairwise sample comparison of metabolomic data for three species was performed using the XCMS online service, providing a direct comparison of two sample groups [21]. From the resulting features of every compared pair of oganisms, we selected only those with a p value≤0.05 in order to state that compound concentration is different in two species. Additionally these compounds were analyzed manually by counting the metabolite identifications among the seven repetitions of every species for both acquisition modes. The quantification of an identified metabolite is possible only if it was detected at least three times in each species. In the case a compound was detected less than three times of seven, only a qualitative comparison in the pair was possible, based on the detection of MS counts for this compound in the pair.

## Results

### MS spectra acquisition

Bacteria were cultivated in liquid media to more easily determine the growth phase. Bacteria were harvested in the logarithmic growth phase, which was determined based on the culture duplication time (see “Bacteria strain and growth conditions”). Cold methanol extraction was used to instantly quench the metabolism [16]. The high effectiveness of this method and its low loss of compounds has been demonstrated previously [22]. Metabolite detection was carried out using HPLC analysis in combination with electrospray ionization quadrupole time-of-flight mass spectrometry (HPLC-MS Q-TOF). The parameters of the chromatographic method, as well as the mass spectrometric peak recording, were optimized using standards to obtain the most reliable data (see “Materials and Methods” section). Metabolites were initially identified using the Metabolomic Analysis and Visualization Engine (MAVEN) software [19]. The metabolite identity was then confirmed by analysis of the detected parent ions fragmentation spectra using targeted mass spectrometry [23].

There were seven independent repeats for metabolite detection for each of the three studied bacterial species. Usually one LC-MS analysis run produces ∼120 peaks in which the m/z values are close to the exact metabolite m/z values (deviating by less than 10 ppm). However, in MS acquisition, some compounds show unstable signals and display low reproducibility; their peak intensities are comparable to the noise level. Each compound was considered to be present in bacteria if it was observed at least three times (in seven runs) in any of the ionization modes (positive or negative) and the intensity of the corresponding peak was significantly higher than that of the background. List of identified metabolites is shown in Table 1.

**Table 1:** Identified metabolites of *S. melliferum*, *M. gallisepticum* and *A. laidlawii*.

Compound name	Compound ID	Detection mode		
		*A. laidlawii*	*S. melliferum*	*M. gallisepticum*
L-Tyrosine	C00082	pos+neg	pos+neg	pos+neg
L-Tryptophan	C00078	pos+neg	pos+neg	pos+neg
L-Aspartate	C00049	neg	pos+neg	pos+neg
L-Serine	C00065	neg	pos+neg	pos+neg
L-Citrulline	C00327			pos+neg
L-Threonine	C00188	pos+neg	pos+neg	pos+neg
L-Proline	C00148	pos+neg	pos+neg	pos+neg
L-Isoleucine/L-Leucine	C00407/C00123	pos+neg	pos+neg	pos+neg
L-Lysine	C00047	pos+neg	pos+neg	
L-Glutamine	C00064		pos	
L-Phenylalanine	C00079	pos+neg	pos+neg	pos+neg
L-Methionine	C00073	pos+neg	pos+neg	pos+neg
L-Arginine	C00062	pos	pos+neg	
L-Histidine	C00135	pos	pos	pos
L-Glutamate	C00025		pos+neg	pos+neg
4-Methyl-2-oxopentanoate	C00233	pos	pos	pos+neg
2–3-Dihydroxy-3-methylpentanoate	C06007	pos	pos	pos
L-Cysteine	C00097		pos	
L-Arogenate	C00826			pos
L-Glycine	C00037	neg	neg	
Mevalonate-5-phosphate	C01107		pos	
S-Ribosyl-L-homocysteine	C03539		pos	pos
S-Adenosyl-L-methionine	C00019	pos		pos
N-Formyl-L-methionine	C03145		neg	neg
5-Oxopentanoate	C03273	pos	pos	
Thymine	C00178	pos	pos	pos
Adenine	C00147	pos+neg	pos+neg	pos+neg
Xanthine	C00385	pos+neg	pos+neg	pos+neg
Hypoxanthine	C00262	pos	pos+neg	pos+neg
Guanine	C00242	pos+neg	pos+neg	pos+neg
Uracil	C00106	pos+neg	pos+neg	pos+neg
Thymidine	C00214	pos+neg	neg	pos+neg
Deoxycytidine	C00881	pos+neg	pos	pos
Cytidine	C00475	pos+neg	pos+neg	pos+neg
Cytosine	C00380	pos	pos	pos
Uridine	C00299	pos+neg	pos+neg	pos+neg
Guanosine	C00387	pos+neg	pos	pos+neg
Adenosine/Deoxyguanosine	C00212/C00330	pos	pos	pos+neg
Deoxyadenosine	C00559	pos	pos	pos
CMP	C00055	pos+neg	pos+neg	pos+neg
dUMP	C00365	pos+neg	pos+neg	neg
UDP	C00015	pos+neg	pos	pos+neg
GMP	C00144	pos+neg	pos+neg	pos+neg
dGMP/AMP	C00362/C00020	pos+neg	pos+neg	pos+neg
dAMP	C00360	pos+neg	pos+neg	neg
UMP	C00105	pos+neg	pos+neg	pos+neg
dGDP/ADP	C00361/C00008	pos+neg	pos+neg	pos+neg
dTMP	C00364	neg	neg	neg
ITP	C00081	pos	pos	pos
dUTP	C00460	pos	pos	pos
2-Deoxyinosine 5-phosphate	C06196	pos		pos
Xanthosine	C01762	pos		neg
Inosine	C00294	neg	neg	
XTP	C00700	neg		neg
dTDP	C00363	neg		neg
dUDP	C01346	neg		
Deoxyuridine	C00526	pos+neg		neg
dTTP	C00459		neg	neg
dCMP	C00239			neg
S-Adenosyl-L-homocysteine	C00021		pos	
a-a-Trehalose/Maltose/Sucrose/Lactose	C01083/C00208/C00185/C00089/C00243		pos+neg	
D-Mannitol/D-Sorbitol	C00392/C00794/C01697	pos+neg	pos+neg	pos+neg
Sedoheptulose 7-phosphate	C05382	neg	neg	neg
D-Ribose 5-phosphate	C00117	neg		neg
GDP-6-deoxy-D-mannose	C03117	neg		pos
D-Mannitol 1-phosphate/D-Sorbitol-1-phosphate	C00644/C01096		pos+neg	
3-Phospho-D-glycerate	C00197			pos
Phosphoenolpyruvate	C00074	neg		neg
N-Acetyl-D-galactosamine 6-phosphate/n-acetyl-D-glucosamine-1-phosphate/N-Acetyl-D-mannosamine 6-phosphat	C06376/C04501/C04257	neg		
Glycerone phosphate/glyceraldehydes-3-phosphate	C00111/C00118		neg	neg
beta-D-Fructose 1–6-bisphosphate	C05378	pos		
Glycerone	C00184	neg	neg	
5-Phospho-alpha-D-ribose 1-diphosphate	C00119	pos	pos	pos
Thiamin diphosphate	C00068	pos	pos+neg	pos+neg
Formate	C00058	neg	neg	neg
Geranylgeranyl diphosphate	C00353	neg	neg	
2-C-Methyl-D-erythritol 2–4-cyclodiphosphate	C11453	neg	neg	neg
5–10-Methenyltetrahydrofolate	C00445	pos	pos	pos
5-Amino-6-(5-phospho-ribosylamino)uracil	C01268	neg	neg	neg
5-Amino-6-(5-phospho-D-ribitylamino)uracil	C04454	neg	neg	
sn-Glycerol 3-phosphate	C00093	pos	neg	pos+neg
2-C-Methyl-D-erythritol 4-phosphate	C11434	neg		
5–10-Methylenetetrahydrofolate	C00143	pos+neg		neg
NADH	C00004	neg		pos
FMN	C00061		pos+neg	pos+neg
FAD	C00016		neg	
Nicotinate D-ribonucleoside	C05841	pos+neg	pos+neg	pos+neg
NAD	C00003		pos+neg	pos+neg
Deamino-NAD+	C00857	pos+neg	pos+neg	pos+neg
Nicotinurate	C05380	pos	pos	pos
2-Acetolactate	C00900	pos	pos	
4-Methyl-3-oxoadipate	C18312	pos		pos
Nitrobenzene	C06813		pos	
UDP-N-acetyl-D-galactosaminuronic acid	C13952			neg
UDP-N-acetyl-D-mannosamine	C01170	neg	pos+neg	pos

Fragmentation spectra were obtained for most of the detected metabolites using targeted mass spectrometry. The fragmentation spectra were compared with standard fragmentation spectra, which were obtained under similar ionization conditions (polarity of ionization and collision energy values were taken into account) and provided by the Metlin MS/MS Spectrum Match service [24], [25], [26]. Figure S1 shows an example of the metabolite identification as provided by the MS/MS. Interestingly, all fragmentation spectra confirmed the primary identification of the compounds based on the m/z values alone. There were no compounds whose fragmentation spectra contradicted the identification based on the m/z values. The provided m/z values usually did not deviate from the theoretical value by more than 10 ppm (see Table 2). Among the detected metabolites, there were several compounds that presented a relatively large deviation from their theoretical ion m/z values (up to 15 ppm m/z). Nevertheless, MS/MS fragmentation spectra render the unambiguous identification of such compounds possible.

**Table 2:** Fragmentation spectra results for the detected metabolites of *S. melliferum*, *M. gallisepticum* and *A. laidlawii*, and fragmentation spectra of the analyzed ion standards from the Metlin database [23] in the same experimental conditions (fixed collision energy of 20 eV).

compound	Ionization Mode	dppm	Parent Mass (*m/z*)	Product Mass (*m/z*) from database Metlin	Found Product Mass (m/z)	Retention Time (minutes)
Adenine	+	13	136.060	136,062;119,035;94,040	136,0575;119,030;94,034	28,5
Thymine	+	12	127.048	127.0502;110.0229;109.0382;84.0439;82.0286;56.0501;54.0340	127.0502;110.0229;109.0382;84.0439;82.0286;56.0501;54.0340	16–17
Hypoxanthine	−	12	135.030	92,025;65,015;66,0097	92,0305;65,0187;66,015	28,3
Uracil	−	8	111.021	41.998	41.999	9
Guanine	+	4	152.056	152,056;135,030;110,035;82,041	152,056;135,030;110,0361;82,038	23,3
Deoxyadenosine monophosphate	+	10	332.072	332.313;136.061;81.0333	332.313;136.061;81.0333	26.3
Guanosine	+	5	284.099	152.0559;135.0286;110.0347	284.0989;152.0559	25
Cytidine	+	7	244.093	112.0492	244.0928;112.0492	25
CMP	+	5	324.059	112.0502	324.0591;112.0502	27
GMP	−	0	362.051	78,959;96,968;150,042;210,999	78,959;96,968;150,042;211,001	38,9
AMP	−	0	346.056	134,047;96,969;78,959	134,0476;96,9698;78,9598	39,2
ADP	−	0	426.022	328,045;276,952;158,924;134,046;78,959	328,0505;276,952;158,9235;134,0475;78,9549	39,5
UDP	−	12	403.000	323,028;111,020;96,969;78,959;	323,028;111,020;96,969;78,959;	24,9
CMP	−	0	322.045	110,033;96,969;78,958	110,025;96,960;78,950	48,4
dTDP	−	14	401.010	−	195,007;78,964;176,997;96,969	39,3
UMP	−	1	323.029	211,001;150,980;138,980;111,0200;96,969;78,959;192,990	211,0014;193,000;150,913;138,980;111,027;96,972;78,959	45,4
dTMP	−	0	321.050	195,005;125,034;78,959;176,995;96,967	195,0089;125,034;78,9584;176,995;96,972	38,8
L-Glutamate	+	10	147.074	102.0549;84,045;56,0502;41,039;85.0291	148.058;102.0549;84,0445;56,0488	5,3
L-Tryptophan	−	0	203.082		142,071;116,055;74,0307	34,5
L-Leucine	+	0	132.102	86,097;44,050;43,055;30,034	86,09499;44,050;43,055;30,034	35,1
L-Isoleucine	+	0	132.102	86,097;57,058;44,050;43,055;30,034	86,0965;57,059;44,051;43,056;30,0345	36,4
L-Cysteine	+	11	122.026	58,996;	58,996;	38,67
L-Threonine	−	2	118.051	74,059;57,035;56,050	74,0601;56,0498	39,0
L-Proline	+	3	116.071	70.066	70.064	34
L-Phenylalanine	+	2	166.086	120,081;103,054	120,0801;103,0539	35
L-Histidine	+	7	156.076	110,071;95,061;93,045;83,061	110,0668;95,0575;81,046	36,2
L-Tyrosine	−	0	180.067	72,0093;93,0343;119,0500;163,038;74,0253	72,010;119,0516;163,0389	47,2
L-Lysine	+	4	147.112	84,081;	84,0766;	36,5
L-Aspartate	−	0	132.030	115,003;88,0401;72,008;71,014;59,0133;42,0328	115,011;88,0454;72,032;71,014;59,0187;42,0384	38,3
L-Serine	−	3	104.036	74.025	74.0272	39,7
L-Arginine	+	8	175.1157	116.0707;70.0658;60.0653	175.1157;70.0658;60.0653	29.2
L-Methionine	+	4	150.057	56.0501;61.0114;104.0524;133.0313;102.0544;74.0244;87.0263	56.0501;61.0114;104.0524	27
D-Glyceraldehyde 3-phosphate/Dihydroxyacetone phosphate	−	3	168.990	96,969;78,959	96,974;78,964	39,4
Sucrose	+	9	365.1022	365.1052;203.0518;185.0415	365.1052;203.0518;185.0415	25.2
2-Acetolactate	−	2	131.035	−	85,8767;45,0019;41,993	32,3
N-Acetyl-D-glucosamine 6-phosphate	−	3	300.050	−	260,387;78,961;32,675;92,923;94,920;96,964	44,8
Sorbitol/Mannitol	−	12	181.075	119,035;101,024;89,024;89,025;73,029;71,0145;59,0144;44,999;43,019	119,050;101,0188;89,018573,025;71,009;59,0140;44,996;43,016	43,05
NADH	+	3–21	665.115	542,062;524,060;428,035;323,084;136,061	542,061;524,059;428,0389;232,084;136,061	38,9
Betaine			118.086	118.086;59.0739;58.0660	118.086;59.0739;58.0660	28–30.7
Cytosine	+	11	112.0489	112.0505;95.0242;94.0401;69.0452;68.0136;67.0296;52.0189	112.0505;95.0242	26.3
NAD	+	3	664.1164	542.0622;524.060;428.035;232.0836;136.0603	664.1164;542.0622;524.060;428.035;232.0836;136.0603	27
dGMP	+	9	386.0227	152.0544;81.033	386.0227;152.0544	24.5

### Metabolite content of *S. melliferum*, *M. gallisepticum*, and *A. laidlawii*

The strategy for metabolome analysis includes the following steps: 1) Metabolic map reconstruction; 2) Prediction of all possible metabolism intermediates; 3) Comparison of metabolic enzymes of Mollicute species; 4) Experimental MS detection and mapping of the detected compounds on the metabolic map for each specie; 5) Juxtaposition of the metabolome data to enzyme differences between three Mollicute species.

**Figure 1:**
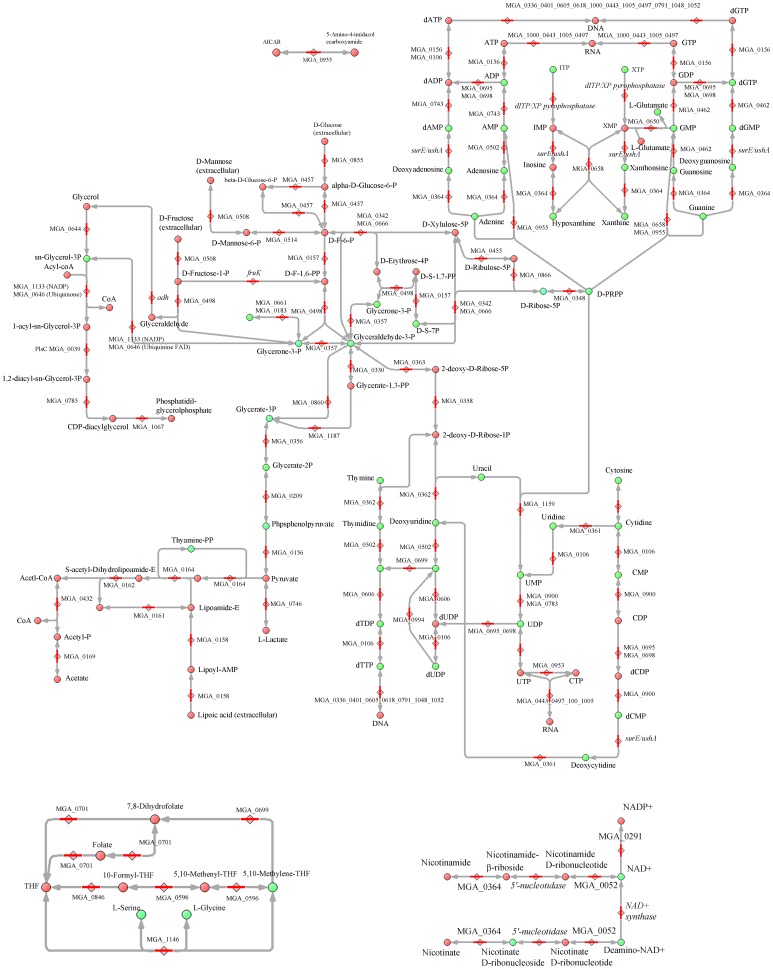
Reconstructed metabolic map of *M. gallisepticum*. The pathways common for three Mollicute species are represented. Metabolites are shown as circles; compounds identified by LC-MS are marked in green, other predicted compounds are marked in red. Proteins that catalyze metabolic reactions are shown as diamonds, and their ID numbers are indicated. Enzymatic activities, which are not associated with annotated proteins, are indicated in italics. Abbreviations: D-F-6-P - D-fructose-6-phosphate; D-F-1,6-PP - D-fructose-1,6-bisphosphate; D-S-1,7-PP - D-sedoheptulose-1,7-bisphosphate; D-S-7-P - D-sedoheptulose-7-phosphate; GAP - glyceraldehyde-3-P).

**Figure 2:**
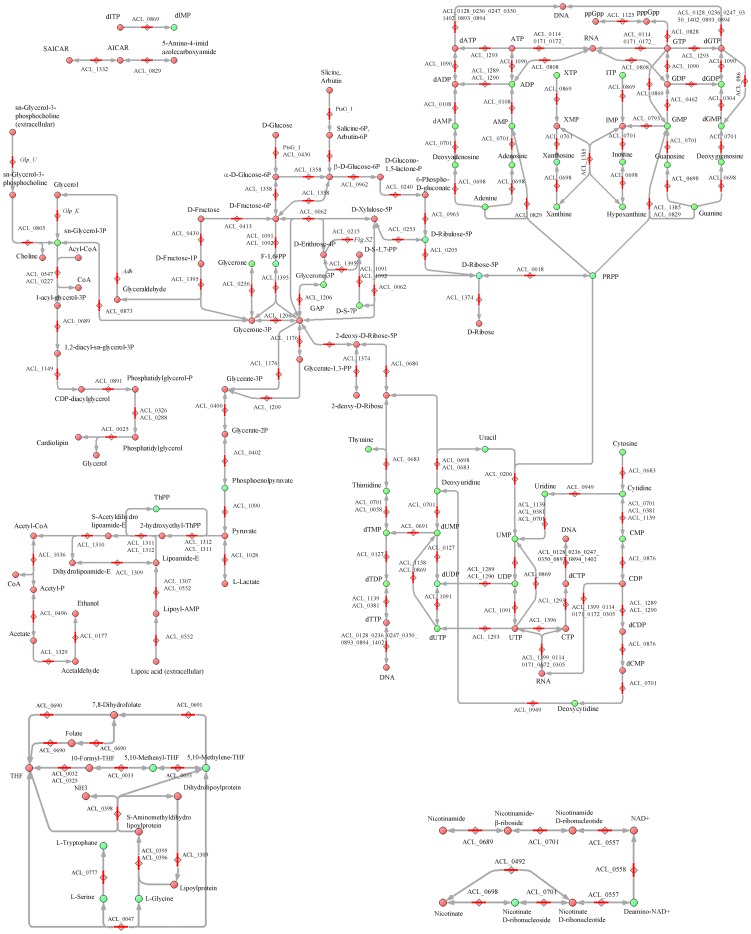
Reconstructed metabolic map of *A. laidlawii*. The pathways common for three Mollicute species are represented. Abbreviations and symbols are given in figure 1.

**Figure 3:**
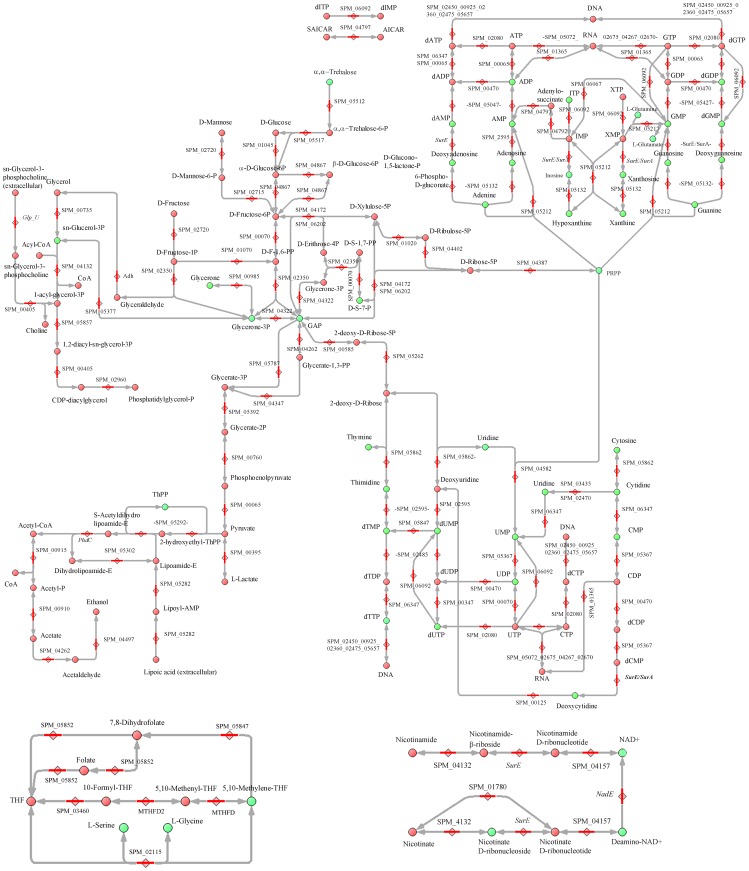
Reconstructed metabolism of *S. melliferum*. The pathways common for three Mollicute species are represented. Abbreviations and symbols are given in figure 1.

We manually reconstructed the metabolic network of *M. gallisepticum*, *S. melliferum*, and *A. laidlawii* in order to fill the network with the detected intermediates. To build a comprehensive metabolic network for each species, we used the Kyoto Encyclopedia of Genes and Genomes database and UniprotKB proteogenomic annotation data provided earlier by our lab [3], [11], [27]. For enzymes that could carry out more than one reaction, we removed the reactions that were decoupled from pathways and those for which the substrate was unavailable (*i.e.* there is no way for substrate synthesis and transport into the cell according to the proteogenomic annotation). The final result is a map without gaps, isolated reactions, or open metabolic loops for each species (Figures 1–3, see text S1 and Figures S2, S3).

For each species a list of predicted metabolism intermediates was formed. The map includes all compounds, which serve as substrates and products for all enzymes and transporters predicted by the proteogenomic annotation. The metabolic database for raw MS data analysis by MAVEN includes all metabolism intermediates predicted for the three species. This database contains 500 items and is suitable for the analysis of each species. The database used is wider than the list of possible metablolites for each species. It therefore permits detection of metabolites produced by putative uncharacterized enzymes.

**Figure 4:**
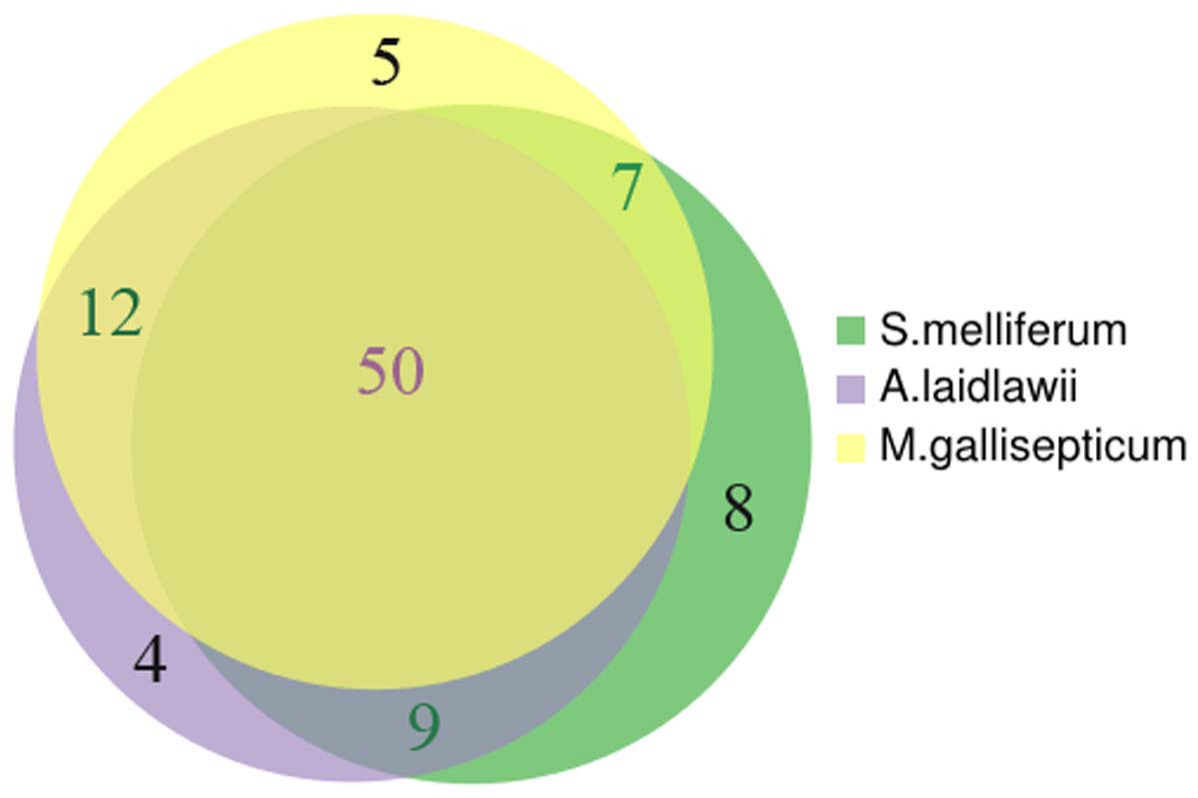
Overlap of the detected metabolites for three Mollicute species. Yellow circle represents metabolites of *M. gallisepticum*, violet circle represents metabolites of *S. melliferum*, and green circle represents metabolites of *A. laidlawii*. The list of identified metabolites of *S. melliferum*, *M. gallisepticum* and *A. laidlawii* is presented in Table 1.

Metabolic pathways were reconstructed with all the identified metabolites of *S. melliferum*, *M. gallisepticum* and *A. laidlawii* positioned in the maps (Figures 1–3). The metabolome predicted by proteogenomic annotation consists of 323 metabolites for *A. laidlawii*, 225 for *M. gallisepticum*, and 361 for *S. melliferum*. We have identified 75, 74 and 74 metabolites for each species, respectively. Accordingly, we were able to detect 20 to 33% of the predicted metabolites by LC-MS using HILIC chromatography (Figure 4 and Table 1).

Reconstruction of the three species' metabolic maps reveals many pathways with enzymatic gaps in the middle. For pathways in which only one enzyme is missing, the gap can be filled by adding an unassigned reaction as performed earlier for another Mycoplasma species, *M. pneumoniae* [23]. Alternatively, for a missing enzymatic reaction, a bypass can be found by the manual analysis of the known enzyme's side activities.

**Figure 5:**
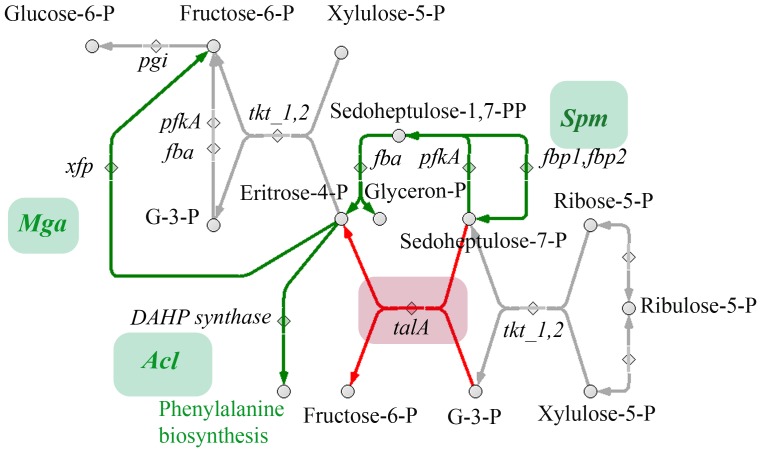
Reactions of the pentose phosphate pathway. Metabolites involved in the pathway are shown as circles; enzymes are shown as diamonds; and the path that we propose to convert sedoheptulose-7-phosphate is marked in green.

One example is the link between pentoses and hexoses (the pentose phosphate pathway) in Mollicutes. This link is omnipresent in living cells, although in many organisms, including mycoplasmas, only its non-oxidative branch is annotated. Two enzymes, transketolase (tktA) and transaldolase (tal), act in this branch of the pentose phosphate pathway (Figure 5). Transketolase transforms two pentoses, xylulose-5-phosphate and ribose-5-phosphate, into the seven-carbon product sedoheptulose-7-phosphate and the three-carbon glyceraldehyde-3-phosphate. To produce the hexose, fructose 6-phosphate, and glyceraldehyde-3-phosphate and to finish the pentose phosphate pathway, the same enzyme uses the four-carbon erythrose-4-phosphate and the pentose xylulose-5-phosphate. A key enzyme, transaldolase, acts to link these two transketolase reactions; it removes three-carbon fragment from sedoheptulose-7-phosphate and condenses it with glyceraldehyde-3-phosphate, forming the fructose 6-phosphate and the erythrose-4-phosphate [28].

Annotation of genome sequence shows that Mollicutes lack transaldolase, and this obviously creates a gap in the metabolic branch. Nevertheless, we detected a unique intermediate of transketolase reaction, sedoheptulose-7-phosphate, in all three species studied (along with less specific intermediates). It suggests that the pentose phosphate pathway is functional. The connection of the glycolytic pathway with a functional pentose phosphate pathway was earlier demonstrated for *M.pneumoniae* as ^13^C-6 glucose carbons were detected in ribose 5-phosphate using mass spectrometry [23].

Database search for transaldolase-like proteins in all other Mollicute genomes produces a negative result. Therefore we believe that transaldolase activity is absent in this genus. To fill the gap, it is possible to postulate the presence of aldolase activity by an unknown mycoplasma protein. The other way is to find a bypass of this reaction performed by other known mycoplasma enzymes. The literature shows that sedoheptulose-7-phosphate can act as acceptor for 6-phosphofructokinase; fructose-bisphosphate aldolase can bind with sedoheptulose-1,7-bisphosphate and decompose it to glycerone phosphate and erythrose-4-phosphate (see Figure 5) [29]. Glycerone phosphate can be isomerized to glyceraldehyde-3-phosphate by triosephosphate isomerase [28].

The proposed modifications in the pentose phosphate pathway make it possible to utilize ribose as an energy source despite the lack of transaldolases. The described pathway is reversible in *S. melliferum*, and *A. laidlawii*. The first reaction, phosphorylation of sedoheptulose-7-phosphate, catalyzed by 6-phosphofructokinase, is irreversible; however, dephosphorylation of sedoheptulose-1,7-bisphosphate can be carried out by fructose -1,6-bisphosphatase [30], annotated in *S. melliferum* [11].

Fructose 1,6-bisphosphatase is not annotated in *A. laidlawii*, and eritrose-4-phosphate, formed as a result of the transketolase reaction between fructose-6- phosphate with glyceraldehyde-3- phosphate, must be utilized in some other way. Eritrose-4-phosphate may be utilized in phenylalanine synthesis, as all enzymes of phenylalanine biosynthesis are annotated in *A. laidlawii* [3]. A lack of fructose-1,6-bisphosphatase, as well as the absence of transaldolase activity in *M. gallisepticum*, was experimentally shown by J.D. Pollack and M.V. Williams [31]. We assume that the resultant eritrose-4-phosphate in *M. gallisepticum* is utilized by D-fructose-6-phosphate D-erythrose-4-phosphate-lyase; this enzyme is not annotated for *M. gallisepticum*, but it is annotated for another Mycoplasma species (*M. fermentans*). We believe that this activity should be assigned for *M. gallisepticum*. This is a way to implement the nonoxidative reactions of the pentose phosphate pathway for *M. gallisepticum*.

Among the detected metabolites we found compounds that participate in the main metabolic pathways, such as glycolysis, amino acid, sugar, and amino sugar metabolism, synthesis of terpenoids, riboflavines, purine and pyrimidine metabolism, etc. We used the LC-MS/MS method to analyze 95 compounds (see Table 1), representing a substantial fraction of the >500 compounds of the total metabolome of three Mollicute species, *M. gallisepticum*, *S. melliferum*, and *A. laidlawii*. These compound were predicted by protein annotation, and performed by our lab in previous studies [3], [11]. In particular, the purine and pyrimidine metabolism maps were the most uniformly and densely filled maps with the identified metabolites (see Figures 1–3). We detected a variety of purine and pyrimidine metabolism products: nitrogenous bases- adenine, thymine, guanine, xanthine, hypoxanthine, uracil, cytosine; their nucleosides and deoxynucleosides and some of their mono-, di-, triphosphate nucleotides and deoxyribonucleotides. It should be noted that we did not consider inosine mono- and dinucleotide, as according to V.M. Boer *et al.*, this compound is not detectable, as it can be confused with peak interference of ADP and AMP [32]. Expression of the annotated proteins that are functionally associated with the metabolism of purine and pyrimidine was demonstrated in our previous studies [3], [11]. In the *S. melliferum* metabolome, we did not detect deoxyuridine, dTDP, XTP, xanthosine, 2-deoxyinosine 5-phosphate; however, these compounds have been detected in *M. gallisepticum* and *A. laidlawii*. Among *M. gallisepticum* and *A. laidlawii* metabolites, we did not identify inosine and dTTP, respectively.

There are many nutrient carriers in the Mycoplasma proteome, so the description of membrane fluxes and the discovery of exogenous metabolites acquired by the membrane transport is an important issue in metabolome research. Carbohydrates and their derivatives (such as glycolysis metabolites; see Figures 1–3) including sedoheptulose-7-phosphate, D-ribose-5-phosphate, D-mannitol 1-phosphate/D-sorbitol 1-phosphate, fructose-1,6-bisphosphate, deoxy-D-ribose-1-phosphate, 5-phospho-alpha-D-ribose-1-diphosphate, 3-phospho-D-glycerate, phosphoenolpyruvate, and n-acetyl-D-galactosamine-6-phosphate/n-acetyl-D-glucosamine-6-phosphate/N-Acetyl-D-mannosamine 6-phosphate were detected in all three species. We detected deoxy-D-ribose-1-phosphate in *M. gallisepticum* and *A. laidlawii* metabolome, while the peak of this compound was considered to be peak interference according to V.M. Boer *et al.* [32]. We detected n-acetyl-D-galactosamine-6-phosphate and beta-D-fructose-1–6-bisphosphate in the *A. laidlawii* metabolome, while 3-phospho-D-glycerate is a characteristic feature of the *M. gallisepticum* metabolome. The latter compound was also detected in *M. pneumoniae* [18]. Detection of phosphorylated sugar products (mannitol 1-phosphate) is in good agreement with proteogenomic annotation [3], [11] (PTS system mannitol-specific (MtlA)-like IIB domain protein MGA_1283) and with the identification of phosphorylated sugars in *M. pneumoniae* [18].

In all three bacteria, using both positive and negative ionization modes, we detected non-phosphorylated forms of mannitol/sorbitol/galactitol (sugars and some amino acids like leucine/isoleucine isomers cannot be differentiated using the HPLC-MS method, since they have particularly identical fragmentation spectra and retention times) and alpha-alpha-trehalose/maltose/cellobiose/sucrose/lactose.

Sugars are the most important energy source for the Mollicutes; their import in the cytoplasm is usually coupled with phosphorylation [11]. Here we directly demonstrate that mycoplasmas contain non-phosphorylated sugars. It was assumed that non-phosphorylated forms of carbohydrates can be accumulated from media or adsorbed to the cell surface [1], [33], [34], [35], [36]. Sugar ABC transporter systems which are not associated with phosphorylation were also annotated (MGA_1076, MGA_1077, MGA_1078 for *M. gallisepticum*, SPM_2645 *S. melliferum*). It cannot be ruled out, but it does seem unlikely that mycoplasmas store sugars to consume them during possible carbohydrate starvation.

We detected 17 of the 20 amino acids in the three Mollicute species. Their presence in the metabolome can be attributed to their exogenous nature and the fact that they are actively imported into the cells through specific transporters (permeases), since no amino acid synthesis enzymes have been annotated in these organisms [11]. Peaks in the MS spectra that corresponded to amino acids have the highest intensity. Only asparagine, alanine, and valine were not detected. In experiments employing the same equipment and another bacteria (not shown), we were able to detect these three amino acids. This suggests that the concentration of the three amino acids, which were not found in *S. melliferum*, *M. gallisepticum* and *A. laidlawii* total metabolome, was significantly lower than the concentration of the seventeen detected amino acids. Of note, in *M. pneumoniae* these three amino acids were detected [18]. Alanine is the most abundant amino acid in proteins and its absence in the metabolome could be explained by its rapid involvement into protein synthesis through aminoacyl-tRNA synthetase.

In *M. pneumoniae* riboflavin was detected by LC-MS [18]. We have detected intermediates of riboflavin biosynthesis, 5-amino-6-(5-phospho-D-ribitylamino)uracil, 5-amino-6-(5-phosphoribosylamino)uracil, FMN and FAD. Among the explored three species, only *A. laidlawii* proteogenomic annotation contains proteins responsible for the diaminohydroxyphosphoribosylaminopyrimidines conversions in addition to the bifunctional FAD synthetase/riboflavin kinase. However, in the *S. melliferum* and *M. gallisepticum* metabolomes 5-amino-6-(5-phosphoribosylamino) uracil mass-spectral peaks were reliably detected with a high intensity. This finding can indicate activity of a metabolic branch responsible for FMN biosynthesis from GTP in *S. melliferum* and *M. gallisepticum*.

## Discussion

The obtained list of metabolism intermediates taken together with our previous studies on the protein annotation of the same cells makes it possible to understand the exact mechanisms of the biochemical reactions in three Mollicute species (*M. gallisepticum*, *S. melliferum*, and *A. laidlawii*). Metabolome studies of the simplest bacteria reveal compounds and pathways that are important for the basic functions of bacteria maintenance. There were some metabolites that were not detected in our experiment but were present in the list of metabolites based on the bacterial proteome [3], [11]. Some metabolites cannot be detected by LC-MS, because, for example, they are unstable under ionization conditions. Metabolites that are non-soluble in methanol cannot be detected either. Another reason why some metabolite intermediates were not detected is their possible consumption during cell wash in poor medium or even due to the residual enzymatic activity during the methanol extraction [22]. We detected several glycolysis intermediates (3-phospho-D-glycerate, phosphoenolpyruvate, glyceraldehyde-3-phosphate, beta-D-fructose 1–6-bisphosphate), so we conclude that the glycolysis rate is significantly decreased under the quenching conditions that we use. Similar sample preparation procedures revealed a similar glycolysis intermediate content in *M. pneumoniae* [18]. Several items that we did not expect to be revealed as metabolism components were detected. For example, in *S. melliferum*, S-ribosyl-L-homocysteine and 5-oxopentanoate were detected. As no enzymes responsible for their utilization or synthesis were annotated, we believe that such compounds are the products of amino acid degradation.

**Figure 6:**
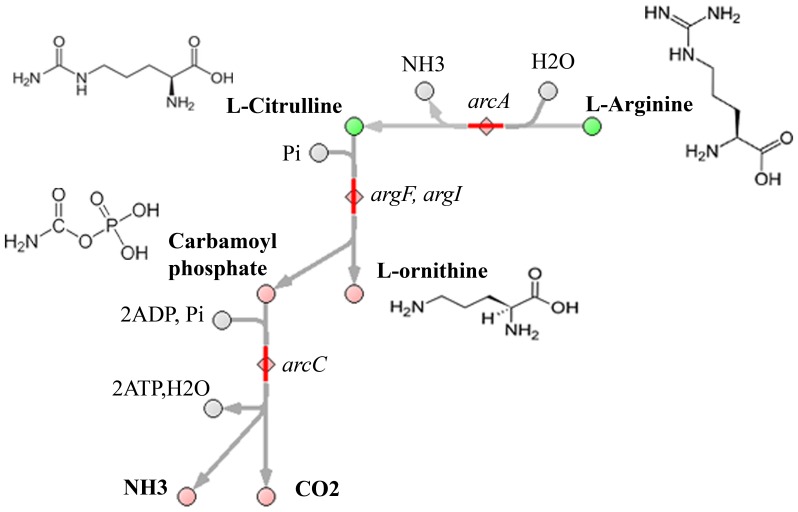
Arginine deiminase (ADI) pathway. In this pathway, metabolites are shown as circles; enzymes are shown as diamonds; and compounds that we detected are marked in green.

The differences that we identified primarily concerned amino acids and lipid biosynthesis and mechanism of carbohydrate assimilation (owing to Mollicutes' parasitic nature, many nutrients are received from the host), and the biosynthesis of terpenoids and riboflavins. We found that citrulline concentration is higher in *M. gallisepticum* compared to *A. laidlawii* and *S. melliferum* while arginine concentration is lower in *S. melliferum* as compared to *A. laidlawii* (Table 3). Citrulline is an intermediate in the arginine deiminase (ADI) pathway. The ADI path converts arginine to citrulline and ammonia, ornithine transcarbamylase subsequently converts citrulline to ornithine and carbamoyl phosphate, and finally carbamate kinase cleaves carbamyl phosphate to ammonia and CO_2_, generating an ATP in that process (see Figure 6). The system also requires an arginine/ornithine antiporter to rid the cell of the end-product, ornithine, while importing a new arginine substrate [27]. Ammonia that is generated by arginine deamination can alkalinize the environment [37], and helps the survival of cultures during acid stress. Some Mycoplasmas (*M. hominis*, *M. arthritidis*, *M. gallinarum* and others) use the arginine pathway only as an alternative energy source [38].The central enzyme of this pathway, arginine deiminase, was identified in *M. gallisepticum* and *S. melliferum* genome and proteome in our previous studies, while it is absent from *A. laidlawii* [3], [11]. Only the *S. melliferum* genome annotation has proteins responsible for providing a complete ADI cycle, including arginine/ornithine antiporter [11], while *M. gallisepticum* lacks ornithine carbamoyltransferase [39].

**Table 3:** Metabolite peaks counting and pairwise comparison of three Mollicute species.

Compound	Compound ID	Spectral counts			Normalized peak intensities ratio	Conclusion
		*M.gal.*	*A.laid.*	*S.melli.*		
L-Lysine	C00047	-	6	10	*S.mellif.*/*A.laid.* = 3.0	*S.mellif.*>*A.laid.*>*M.gal*. = 0
L-Glutamate	C00025	5	-	4	*S.mellif.*/*M.gal. = 1.2*	*S.mellif.* = *M.gal.>A.laid.* = 0
L-Glutamine	C00064	-	-	4	-	In *S.mellif.* only
CMP	C00055	8	7	12	*S.mellif.*/*M.gal.* = 3.7 *S.mellif.*/*A.laid.* = 1.6 *A.laid.*/*M.gal.* = 1.8	*S.mellif.*>*A.laid.*>*M.gal.*
sn-Glycerol 3-phosphate	C00093	11	7	4	*A.laid./S.mellif.*/ = 1.4 *M.gal./A.laid.* = 24 *M.gal./S.mellif.* = 53	*M.gal.*>*A.laid.*≥*S.mellif.*
5-Amino-6-(5-phospho-D-ribitylamino)uracil	C04454	-	6	-	-	In *A.laid.* only
5-Amino-6-(5-phosphoribosylamino)uracil	C01268	3	7	7	*A.laid.*/*M.gal.* = 5.4 *S.mellif*/*A.laid* = 2.6 *S.mellif*/*M.gal.* = 30	*S.mellif.*>*A.laid.*>*M.gal.*
2-C-Methyl-D-erythritol 4-phosphate	C11434	-	6	-	-	In *A.laid.* only
2-C-Methyl-D-erythritol 2–4-cyclodiphosphate	C11453	7	6	4	*M.gal.*/*A.laid.* = 9.1 *M.gal.*/*S.mellif.* = 20 *A.laid.*/*S.mellif* = 1.3	*M.gal.*>*A.laid.≥S.mellif.*
L-Arginine	C00062	-	5	4	*A.laid.*/*S.mellif.* = 2.2	*A.laid.*>*S.mellif.>M.gal = 0*
L-Citrulline	C00327	11	-	-	-	In *M.gal.*only
Geranylgeranyl diphosphate	C00353	0	7	5	*A.laid.*/*S.mellif.* = 3.3	*A.laid.*>*S.mellif.>M.gal = 0*
a-a-Trehalose/Maltose/Sucrose/Lactose	/C00208/C00185/C00089/C00243	-	-	5	-	In *S.mellif.*only

Results are summarized for the positive and negative acquisition modes.

Another way that microbes use to respond to acidification is by producing enzymes that can convert acidic metabolites to neutral products or neutral metabolites to alkaline products. Good examples of this type of enzymes are glutamate decarboxylase, lysine decarboxylase, and arginine decarboxylase of *Escherichia coli*, all of which exhibit increased expression at external acidic pH [40]. Ammonia can also be produced by the degradation of L-histidine, such as by the activity of L-histidine ammonialyase detected in ureaplasmas [41]. Glutamate decarboxylase is absent from all Mycoplasma species. The *A. laidlawii* annotation lacks arginine deiminase, glutamate decarboxylase or arginine decarboxylase, while the lysine decarboxylase annotated for *A. laidlawii* is unique among all Mollicutes. Lysine is a basic amino acid and possesses two amino groups. We found that lysine concentration is decreased in *A. laidlawii* compared to *S. melliferum*. Lysine decarboxylation can possibly serve as a mechanism to aid the survival of cultures in acid extracellular conditions.

Host infection caused by bacteria of both species, *S. melliferum* and *M. gallisepticum*, necessitates acid adaptation of the pathogens. When infected, chickens and turkeys mycoplasmosis bacteria *M. gallisepticum* should adapt to acidic environment conditions. The clinical manifestation following the infection is called chronic respiratory disease in chickens and infectious sinusitis in turkeys [26]. As the infection begins with the colonization of the respiratory tract, tracheitis and airsacculitis are the predominant symptoms of a localized infection in chickens [42]. In turn, bees are the main reservoir for spiroplasmas, where they primarily invade the gut lumen. Some species expanded their habitat range to include hemolymph, ovaries, fat bodies, hypodermis, and salivary glands. Spiroplasma infection of bees occurs with nectar consumption. The invertase of the pharyngeal gland secretions inverts sucrose into glucose and fructose, and then glucose oxidase converts glucose into gluconic acid to acidify honey (pH 4–5). To survive, spiroplasmas need to overcome this pH-barrier. The balance of *citrulline/arginine* during the growth of Mycoplasmas will be investigated in future research.

We suggest that *A. laidlawii* has adaptation mechanisms different from *M. gallisepticum* and *S. melliferum* and uses decarboxylation of lysine since *A. laidlawii* is widely distributed in nature and has infects a wide range of hosts [43]. Members of the *Acholeplasma* genus do not have specific host associations and have been found in almost every type of living organism. They are one of the five common reasons of cell culture contamination, and a causative agent of some plant diseases [44], [45], [46], [47].

To check whether *M. gallisepticum*, *A. laidlawii* and *S. melliferum* are able to survive in acidic environment, we used the “color test” method to estimate a fraction of the surviving cells and found that *M. gallisepticum*, *A. laidlawii* and *S. melliferum* can survive at low values of pH (∼5.0–5.3); this type of adaptation can be necessary during an infection.

We detected intermediates of purine metabolism (Figures 1–3). It should be noted that a direct transition between purine nitrogen base and monophosphate nucleotide is catalyzed by AMP pyrophosphorylase, which is found in all three species. Sequential conversion of nucleotides- to nucleosides and then to nitrogen bases is performed by the protein 5′-nucleotidase. This protein is not annotated for *S. melliferum* (in *A. laidlawii*, this protein is annotated, while *M. gallisepticum* contains the protein thymidine kinase, which is capable to catalyzing this reaction). Purine nucleoside phosphorylase is available in all three species. 5′-nucleotidase makes it possible to 1) consume AMP and use it as an energy source; 2) to use ribose from AMP as a building block for other nucleotide synthesis. However, we do not believe that 5′-nucleotidase should be assigned for *S. melliferum*, as was suggested for *M. pneumoniae* [23]. We believe that the purine metabolism pathway in *S. melliferum* is directed from phosphoribosyl-pyrophosphate to DNA and RNA synthesis while the reverse direction is absent (*S. melliferum* does not utilize nucleotides).

We detected a high level of glycerol-3-phosphate in *A. laidlawii* and a decreased CMP level compared to *S. melliferum* and *M. gallisepticum* (see Table 3). The composition of the *A. laidlawii* membrane differs from that of other Mollicutes, since the main lipids of its cytoplasmic membrane are the neutral glycolipids monoglucosyl diacylglycerol (MGDG) and diglucosyl diacylglycerol (DGDG), the phosphoglycolipid glycerophosphoryl diglucosyl diacylglycerol (GPDGDG), and the phospholipid phosphatidylglycerol (PG), whereas cholesterol is a major membrane component of other mycoplasmas [8]. An important reaction in phospholipid synthesis is the replacement of the cytosine unit by glycerol-3-phosphate to form phosphatidylglycerol (PG) and CMP. PG is a precursor of lipoglycans, the main component of the *Acholeplasma* membrane. Glycerol kinase is absent from the *A. laidlawii* genome annotation, while glycerol-3-phosphate (G3P) dehydrogenase is present [3]. G-3-P dehydrogenase catalyzes the reversible redox conversion of dihydroxyacetone phosphate to sn-glycerol 3-phosphate and serves as a major link between carbohydrate metabolism and lipid metabolism. A decreased level of glycerol-3-phosphate and a high content of CMP indicate an active synthesis of the main structural component of biological membranes - glycerophospholipids in *A. laidlawii*.

**Figure 7:**
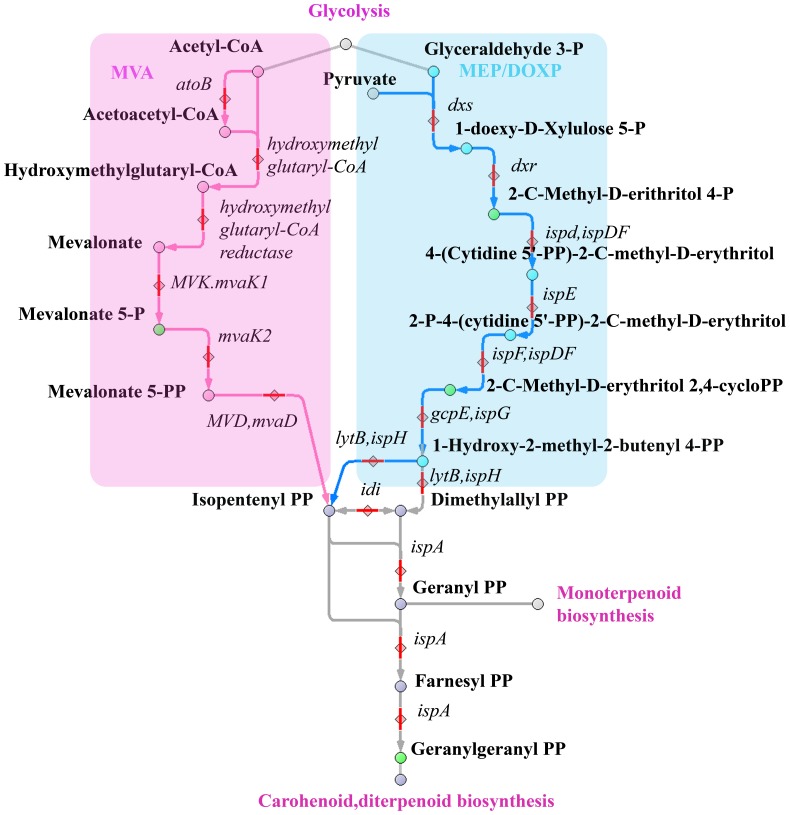
Terpenoid backbone biosynthesis pathway. Metabolites involved in this pathway are shown as circles; enzymes are shown as diamonds; and compounds that we detected are marked in green.

There are two metabolic pathways of terpenoid synthesis: the mevalonate acid pathway (MVA) and nonmevalonate pathway (2-C-methyl-D-erythritol 4-phosphate/1-deoxy-D-xylulose-5-phosphate pathway (MEP/DOXP pathway)) (Figure 7). Although both pathways, MVA and MEP/DOXP, are mutually exclusive in most organisms, interactions between these pathways have been reported in plants and several bacterial species [48], [49]. Many organisms manufacture terpenoids through the mevalonate acid pathway (MVA), that also produces cholesterol. These reactions, directed to carotenoid biosynthesis, take place in the *A. laidlawii* [3]. We have detected a high level of 2-C-methyl-D-erythritol 4-phosphate in *A. laidlawii*, we have not detected any metabolites involved the MVA path.

The alternative nonmevalonate pathway or 2-C-methyl-D-erythritol 4-phosphate/1-deoxy-D-xylulose-5-phosphate pathway (MEP/DOXP pathway) of isoprenoid biosynthesis is an alternative metabolic pathway leading to the formation of isopentenyl pyrophosphate (IPP) and dimethylallyl pyrophosphate (DMAPP). For numerous microbial pathogens such as *Enterobacteria*, *Mycobacterium tuberculosis*, *M. gallisepticum* and *S. melliferum*, the nonmevalonate pathway is the exclusive source of terpenoids [49]. Because the enzymes of the nonmevalonate pathway have no orthologs in mammalian hosts, they are attractive targets for the development of novel antibiotic or antiprotozoal agents. In *M. gallisepticum and S. melliferum* metabolome, we detected the presence of substantial amounts of 2-C-methyl-D-erythritol-2,4-cyclodiphosphate (component of the MEP/DOXP branches of metabolism), and found geranylgeranyl diphosphate (an important precursor in the biosynthesis of all higher terpenoids and carotenoid biosynthesis) only in *S. melliferum*. In *M. gallisepticum* and *S. melliferum* metabolisms, the presence of MEP/DOXP pathway proteins can be found, but the path terminated at the 1-hydroxy-2-methyl-2-butenyl 4-diphosphate [11] (Whole Genome Shotgun ID: AFFR00000000). Nevertheless, the identification of MEP/DOXP pathway components in the metabolome suggests active expression of annotated proteins and terpenoid backbone biosynthesis in *M. gallisepticum* and *S. melliferum*. Interestingly, we have also detected mevalonate-5-phosphate in *S. melliferum* metabolome; however, no proteins of the mevalonate acid pathway are noted in these bacteria.

We have systematically studied three bacterial species that belong to the class Mollicutes: the smallest and simplest bacteria, *Spiroplasma melliferum*, *Mycoplasma gallisepticum*, and *Acholeplasma laidlawii*. We described the basic difference in the principles of three species' metabolic adaptation to acidic environmental conditions and analyzed three species' their metabolomes to confirm our assumptions. The metabolic pathways of three species were reconstructed and analyzed using the proteogenomic annotation data provided by our lab in previous studies [3], [11].

## Supporting Information

Text S1 **Metabolome reconstruction.**
(DOCX)

Figure S1 **MS/MS Spectrum match of Uracil fragmentation spectrum obtained for the** ***S. melliferum*** **sample.** Uracil fragmentation spectrum obtained for the *S. melliferum* sample is above the OX axis and standard Uracil fragmentation spectrum in positive ionization mode collision energy 20 eV from Metlin Metabolites database is under the OX axis [30]; Uracil M+H: m/z = 113.03344, Δ = 10 ppm.
(TIF)

Figure S2 **Reconstructed pathways characteristic for** ***A. laidlawii*** **which are absent in** ***M. gallisepticum*** **.** For description see legend to Figure 1.
(TIF)

Figure S3 **Reconstructed pathways characteristic for** ***S. melliferum*** **which are absent in** ***M. gallisepticum*** **.** For description see legend to Figure 1.
(TIF)
