# Supplementary material for: Metabolomic Analysis of Three Mollicute Species
Source: PLoS One. 2014 Mar 4;9(3):e89312. doi: 10.1371/journal.pone.0089312 (PMC3942410; doi:10.1371/journal.pone.0089312)
Supplement: Text S1 — Metabolome reconstruction. (DOCX) [file pone.0089312.s001.docx]

Supplementary materials

**SUPPLEMENTARY TEXT S1**

Metabolome reconstruction

1. **Abolished reactions**

*Mycoplasma gallisepticum* annotation (in contrast to *Acholeplasma laidlawii* and *Spiroplasma melliferum*) contains mannitol transport protein (PTS system mannitol-specific (MtlA)-like IIB domain protein, MGA_1283) while the mannitol-1-phosphate 5-dehydrogenase protein, which provides a succeeding reaction with mannitol consumption, is absent. We suppose that MGA_1283 doesn’t take part in mannitol transport to the cells [1].

Among annotated proteins of *M. gallisepicum, A.laidlawii* and *S. melliferum*  we found phosphomannomutase (MGA_0358, SPM_05262, ACL_0849|pmm), which catalyzes the isomerizetion of mannose-6-phosphate to mannose 1- phosphate, is not used in any other reaction [2,3,4]. Hence, we removed this reaction from the mannose metabolism branch, because the phosphomannomutase generates a dead end. It should be noted that in closely related species, such as *A.laidlawii*, *deoB* (locus ACL_0680) is annotated as phosphopentomutase (*ppm*), a missing enzyme in the *M. gallisepticum* and *S. melliferum*. We designate the phosphopentomutase catalyzing reaction transition of 2-deoxy-D-ribose 1-phosphate to 2-deoxy-D-ribose 5-phosphate in *M. gallisepticum* and *S. melliferum*.

1. **Dead end solutions. Reactions catalyzed by major proteins**

The mannose consumption path in Mollicutes consists of successive steps involving mannose uptake and concomitant phosphorylation to mannose 6- phosphate (PTS mannose-specific system); isomerization of that ester to fructose 6-phophate (Mannose-6-phosphate isomerase) and phosphorylation of fructose 6-phophate by ATP to fructose 1,6-bisphophate and ADP (fructose 6-phosphate kinase). In *M. gallisepticum,* and *S. melliferum* all concerned proteins are annotated, except PTS mannose-specific system proteins. However, the mannose uptake by *M. gallisepticum* is proved by Rottem S. and S. Razin [5]. These data indicate that the mannose transporter in *M. gallisepticum* is present. We supposed that mannose is taken up by the fructose permease FruA as seems to be the case in other bacteria [6].

Another dead end is phosphorylating glycerone, a metabolite that is not the product of any other reaction, but is generated spontaneously upon hydrolysis of glycerone phosphate and is very toxic [7,8].

In *M. gallisepticum* and *S. melliferum*  purine and pyrimidine metabolism the transition between AMP and Adenosine is absent. Usually this reaction is induced by 5’-nucleotidase, which present in *A.laidlawii* annotation and many other Mollicutes [9]. Although this transition can be implemented in thymidine phosphorilation by the thymidine kinase (MGA_0502), which catalyses this reaction as well as those of adenylate-nucleoside phosphotransferase [10].

1. **Annotation gaps. Assigned proteins**

The fructose active transport has been previously demonstrated in experiments with labeled compounds [5]. In Mollicute cells the fructose-1-phosphate (the product of fructose transport into the cell by PTS fructose-specific system) can be decomposed to glycerone-3-phosphate and glyceraldehyde by fructose-bisphosphate aldolase. The first product of fructose-6-phosphate decomposition — glyceron phosphate — is immediately utilized. The second is glyceraldehyde converted in Mollicutes to the glycerol by alcohol dehydrogenase, which is annotated in *A.laidlawii* and *S. melliferum* proteogenome (SPM_04497, ACL_0177, ACL_0420)[2,3], but absent in the *M. gallisepicum* annotation [4]. In addition, fructose 1-phosphate can be phosphorylated to the fructose 1,6-bisphosphate by the 1-phosphofructokinase fructose 1-phosphate kinase annotated for *S. melliferum*  (SPM_01070). Based on the fact that fructose is actively consumed by the *M. gallisepicum* cells and relatives in *A.laidlawii, S. melliferum* and *M. pneumoniae* proteogenomic annotation proteins of fructose metabolism are present [2,3,8], we marked both path proteins on the *M. gallisepicum* metabolic map.

Mollicutes dUTP / dUDP transition is catalyzed by uridine kinase (MGA_0106, ACL_1139, SPM_05627), while the uridine phosphorylation is carried. In purines and pirimidines metabolism of *M. gallisepicum* some proteins that catalyze several reactions (for example *deoA, deoB,surE/ushA,* *dITP/XTP pyrophosphatase*) are absent, although they were annotated for *S. melliferum* and *A.laidlawii* and metabolome data confirm the presence of their intermediates in *M. gallisepicum*  metabolome. In such cases, the presence of these proteins is obvious, so we assigned proteins in *M. gallisepicum*  metabolism map.

1. **Metabolite accumulation analysis for three species**

To determine the difference in the metabolism management in three Mollicute species we analyzed intermediates’ accumulation for several metabolic pathways to determine intermediates of highest quantity. Metabolite accumulation hints that its reaction represents a limiting stage of the metabolic pathway. The differences in the metabolic nodes between species makes it possible to understand the global direction of catabolic and existing partial anabolic mechanisms as well as to elucidate the difference in how three species globally manage the supply and demand of the basic building compounds of the cell. For different nodes, we examined the corresponding proteins and compared their sequences for three species to find amino acids that could affect their enzymatic activities.

For this purpose we have divided reconstructed maps into the simplest lines (modules) so, that each linear pathway module does not interconnect with other one. For example, the purine metabolism was divided into the following lines:

- PRPP-Adenine- Deoxyadenosine- dAMP- dADP- dATP
- PRPP-Adenine- Adenosine- AMP- ADP- ATP
- Hypoxanthine- Inosine- IMP- ITP
- Hypoxanthine- IMP
- Xanthine- XMP
- Xanthine- Xanthosine- XMP-XTP
- XTP-GMP
- PRPP-Guanine- Guanosine-GMP-GDP-GTP
- L-Glutamine-L-Glutamate
- GDP-dGDP
- PRPP-Guanine- Deoxyguanosine-dGMP-dGDP-dGTP

We then quantitatively compared the integrated intensity of metabolites in each module. We identified compounds with maximal intensity, or "nodes," for each module. Most of the nodes were same, while some nodes were different in *S. melliferum*, *M. gallisepticum*, and *A. laidlawii.* For different nodes we selected the proteins, responding to its utilization or synthesis and analyzed sequences of these proteins for three species (see table S1).

In the purine metabolism, the general major nodes for all three species are the following nitrogenous bases: adenine, guanine, xanthine, hypoxanthine, GMP and L-glutamate, (glutamate formation is accompanied the transition of XMP to GMP). In all three species adenine is the predominant nitrogen base, but in *M. gallisepticum* nitrogen bases the guanine pool is also highly represented. This could be expected, as the Mollicutes genome is AT-rich, and adenines are the most frequently consumed among purines and pirimidynes.

In *S. melliferum* «PRPP – Guanine – Deoxyguanosine – dGMP – dGDP - dGTP» module, we revealed an accumulation of dGMP and deoxyguanosine, while in both *M. gallisepticum* and *A. laidlawii*, deoxyguanosine and guanine are equally present. In modules «PRPP-Adenine-Adenosine-AMP-ADP-ATP» and «PRPP-Guanine-Guanosine-GMP-GDP-GTP» we noted two almost equally paired nodes: Adenine = AMP and Guanine = GMP for *S. melliferum*, while in *M. gallisepticum* and in *A. laidlawii* purine bases are more presented than monophasphates.

In pyrimidine metabolism, cytidine is mainly considered to be one of the key nutrients for the other nucleotide synthesis for Mycoplasmas [8]. Cytosine-Cytidine-Uridine-UMP-UDP-UTP module the following accumulated intermediates are: in *A. laidlawii,* UDP> UMP; in *S. melliferum,* Cytosine = UMP; in *M. gallisepticum,* uridine.

Thymidine is accumulated in *A. laidlawii*. dTMP and dTTP are accumulated in *S. melliferum.* In the thymidine module of *M. gallisepticum* metabolism there are no intermediates accumulation, which suggests that the rate of thymidine utilization in the *M. gallisepticum* is high.

The glycerol import is a prerequisite for the synthesis of glycerophospholipids required to build the membranes. In the chain «Glycerol-sn-Glycerol 3-phosphate-1-Acyl-sn-glycerol 3-1,2-Diacyl - CDP- diacylglycerol -Phosphatidylglycerophosphate » in all three Mollicutes species glycerol 3-phosphate is the maximum, as well as in the chain « Glycerol-Glycerol 3-phosphate-D-Glycerone-phosphate-Glyceraldehyde 3-phosphate ». Glycerol phosphorilation takes place during its transport through the membrane by Glycerol kinase, followed by acetylation of the glycerol 3-phosphate or its oxidation to glycerone-phosphate by the glycerol 3-P oxidase and its use as an energy source. We hypothesized that the glycerol -3-phosphate acetylating protein exhibits reduced activity; in *A. laidlawii* glycerol -3-phosphate accumulation approximately equal to one of the glycerone-phosphate/ glyceraldehyde 3-phosphate (they are isomers and have similar chromatography-mass spectrometric parameters, so it’s difficult to find the difference between them using the LC-MS/MS method). Such features can be associated with a faster recovery of glycerol 3-phosphate and higher content of glycero-phospholipids in the membrane.

Of particular interest were the metabolite accomulations in the sugars catabolism, specifically in glycolysis and PPP. We identified nodal differences in the *A. laidlawii* from the *S. melliferum* and *M. gallisepticum*. While *S. melliferum* and *M. gallisepticum* accumulate Glyceraldehyde 3-phosphate / Glycerone phosphate, the main nodes in the sugars catabolism of *A. laidlawii* are D-fructose-phosphate / D-glucose-phosphate and phospho-D-glycerate and pair of two equal nodes sedoheptulose 7-phosphate and D-ribose 5-phosphate.

1. **Sequence Alignments**

We performed multiple alignments of the analyzed proteins *S. melliferum*, *M. gallisepticum*, and *A. laidlawii* with *E. coli* and *B. subtilis* homologues as reference using the ClustalW algorithm on the web service <http://www.ebi.ac.uk/Tools/msa/clustalw2/>. We used the homologous proteins from other organisms if the crystal structures of *E. coli* and *B. subtilis* were absent in the databases. We used the PDB (<http://www.rcsb.org/pdb/home/home.do>) database for amino acid substitution’s analysis in the active center of analyzed proteins.

Amino acid substitutions in the active sites of the analyzed enzymes could lead theoretically to a difference in the amounts of the analyzed metabolites in the different bacteria. However we need to have a quantitative proteomics data about the all analyzed proteins for the complete understanding of the impact of amino acid substitutions on enzyme activity in all three species.

- 1. **DeoD. Purine nucleoside phosphorylase DeoD-type**

There are no significant amino acid substitutions in the active site. There is a duplication of the gene in *S. melliferum.*

tr|Q7NAP9|Q7NAP9_MYCGA -MTPHIKAK-KEEIAKTVIMPGDPLRAKWIAETFLEDYKLVNEVRNMFAY 48

tr|G9Y037|G9Y037_SPIME -MTPHINAK-KEEIAKVVLMPGDPLRAKTVAEKYLTNLKLVNEVRNMLMY 48

tr|A9NG34|A9NG34_ACHLI MATPHIELQDKSLIAKTVLMPGDPLRAKFIAETYLKDVVQINGVRNMLGY 50

sp|O34925|DEOD_BACSU -MSVHIGAE-KGQIADTVLLPGDPLRAKFIAETYLENVECYNEVRGMYGF 48

sp|Q81T09|DEOD_BACAN -MSVHIEAK-QGEIAESILLPGDPLRAKYIAETFLEDVTCYNNVRGMLGF 48

sp|P0ABP8|DEOD_ECOLI MATPHINAE-MGDFADVVLMPGDPLRAKYIAETFLEDAREVNNVRGMLGF 49

: ** : :*. :::******** :**.:* : * **.* :

tr|Q7NAP9|Q7NAP9_MYCGA TGKYKNKLVTVMGHGMGNPSIGIYTHELYNFYDVENIIRVGSCGALVKDV 98

tr|G9Y037|G9Y037_SPIME TGKYKNIEVTIAGSGMGCPSIGIYSYELFKFYDVDYIIRIGSAGSYNPNI 98

tr|A9NG34|A9NG34_ACHLI TGTYKGRRISVMGSGMGMPSIGIYSYELFKFYDVENIIRIGSAGAYTDKL 100

sp|O34925|DEOD_BACSU TGTYKGKKISVQGTGMGVPSISIYVNELIQSYDVQNLIRVGSCGAIRKDV 98

sp|Q81T09|DEOD_BACAN TGTYKGKRVSVQGTGMGVPSISIYVNELIQSYGVKNLIRVGTCGAIQKDV 98

sp|P0ABP8|DEOD_ECOLI TGTYKGRKISVMGHGMGIPSCSIYTKELITDFGVKKIIRVGSCGAVLPHV 99

**.**. ::: * *** ** .** ** :.*. :**:*:.*: .:

tr|Q7NAP9|Q7NAP9_MYCGA NLGDVILESKAYSESPYAMLIGVDVVDKTNYPSAKLLELSRKTADELKIK 148

tr|G9Y037|G9Y037_SPIME KVYDIFNVREAFGESNYAK-IAANIDSNLIAASEKLFNEIEAVAQAKGIK 147

tr|A9NG34|A9NG34_ACHLI NLYDVVIAKNCWSESSYAKTMGVSGRKKLS-ATKKLNTKLKNTAKRLGIP 149

sp|O34925|DEOD_BACSU KVRDVILAMTSSTDSQMNR-VAFGSVDFAPCADFELLKNAYDAAKDKGVP 147

sp|Q81T09|DEOD_BACAN KVRDVIIAMTACTDSNMNR-LTFPGFDFAPAANFDLLKKAYDAGTEKGLH 147

sp|P0ABP8|DEOD_ECOLI KLRDVVIGMGACTDSKVNR-IRFKDHDFAAIADFDMVRNAVDAAKALGID 148

:: *:. . :* : . . .: .. :

tr|Q7NAP9|Q7NAP9_MYCGA YHEGLVICEDAFYQT-LYTPKQANEKWNAIAVEMEGFALNANAQKAKKHA 197

tr|G9Y037|G9Y037_SPIME THTGIAHAADVFYR--YDDSLDFIHTHNLDVVEMESYALFTNAIVTQKHA 195

tr|A9NG34|A9NG34_ACHLI MTEGTIHSSDVFYRINGDEYKDIYEKHGALCVEMESFALFANALATGKNA 199

sp|O34925|DEOD_BACSU VTVGSVFTADQFYND-DS-QIEKLAKYGVLGVEMETTALYTLAAKHGRKA 195

sp|Q81T09|DEOD_BACAN VRVGNVLTADVFYRE-SMDMVKKLGDYGVLAVEMETTALYTLAAKYGVNA 196

sp|P0ABP8|DEOD_ECOLI ARVGNLFSADLFYSP-DGEMFDVMEKYGILGVEMEAAGIYGVAAEFGAKA 197

* * ** . . **** .: * :*

tr|Q7NAP9|Q7NAP9_MYCGA MTILTVSDSLVTHESMSPEKRQTTFKNMMELALNVANKL--- 236

tr|G9Y037|G9Y037_SPIME ATLLTVSDHLVTKEVTTALERQNNFMTMVELALDTAVQLMG- 236

tr|A9NG34|A9NG34_ACHLI ACILTISDSLVTHEATSAEERQNAFTKMMEIALNTKL----- 236

sp|O34925|DEOD_BACSU LSILTVSDHVLTGEETTAEERQTTFHDMIEVALHSVSQ---- 233

sp|Q81T09|DEOD_BACAN LSVLTVSDHIFTGEETTSEERQTTFNEMIEIALDAAIQQ--- 235

sp|P0ABP8|DEOD_ECOLI LTICTVSDHIRTHEQTTAAERQTTFNDMIKIALESVLLGDKE 239

: *:** : * * :. :**. * *:::**.

- 1. **Hpt. Hypoxanthine-guanine phosphoribosyltransferase**

We found (relative to *E. coli*) I158F substitution in G9XZJ2_SPIME, N106K substitution in G9XX66_SPIME and G9XZJ2_SPIME for *S. melliferum*, and N106A substitution in A9NE16_ACHLI for *A. laidlawii*.

tr|Q7MBB3|Q7MBB3_MYCGA MSKLNYTIEKILITNEQINEAAIKAANWINQTY------KDDEIILVGIL 44

tr|G9XX66|G9XX66_SPIME -MKIHPLVKEILITTEEIDEKCFELGQQISHYYLNEYPAKDNTILCLGLL 49

tr|A9NE16|A9NE16_ACHLI ---MHQDILEVLVSESQIKEITEKLGKQLTHDY------AGKNALFVGLL 41

sp|P37472|HPRT_BACSU --MMKHDIEKVLISEEEIQKKVKELGAELTSEY------QDTFPLAIGVL 42

sp|P0A9M2|HPRT_ECOLI ---MKHTV-EVMIPEAEIKARIAELGRQITERYKD----SGSDMVLVGLL 42

tr|G9XZJ2|G9XZJ2_SPIME ---MQTDKLELYLSEDKIQDKIKDYAEKINKLY------AGKKLYCIGLL 41

:: :: :. :*. . . :. * . :*:*

tr|Q7MBB3|Q7MBB3_MYCGA KGCIPFIGKIIDKIE-CEMILDFMTLSSFKGETKSQGTPKIVMDLAYDVM 93

tr|G9XX66|G9XX66_SPIME KGCIPFMAKFIAHLVGVECETEYMVVSSYFGGVKSKDTPQILLDLPMPIT 99

tr|A9NE16|A9NE16_ACHLI NGCVPFMSELVLKID-THIEIQFMAVSSYHGGIKSSGDVKIKYDLNSPVQ 90

sp|P37472|HPRT_BACSU KGALPFMADLIKHID-TYLEMDFMDVSSYGNSTVSSGEVKIIKDLDTSVE 91

sp|P0A9M2|HPRT_ECOLI RGSFMFMADLCREVQ-VSHEVDFMTASSYGSGMSTTRDVKILKDLDEDIR 91

tr|G9XZJ2|G9XZJ2_SPIME NGALFFMSDLLKQLK-VQIVIDTMNISSYIG-TQSTNKITIHKDISKDII 89

.*.. *:..: .: : * **: . : * *: :

tr|Q7MBB3|Q7MBB3_MYCGA DKNVLLVEDIIDSGNTIKIVLDLLKQRGAKSVRLLTFLDKPSGREVDVKA 143

tr|G9XX66|G9XX66_SPIME NRDILLVEDIIDSGKTIKMIKDYLYLKGARSVKVVTLLDKKAGRKVDLVA 149

tr|A9NE16|A9NE16_ACHLI DRDVVIVEDIVDTGATLKTVTELLKYRGAKSVKVVTLLDKPSGRKVDFIP 140

sp|P37472|HPRT_BACSU GRDILIIEDIIDSGLTLSYLVELFRYRKAKSIKIVTLLDKPSGRKADIKA 141

sp|P0A9M2|HPRT_ECOLI GKDVLIVEDIIDSGNTLSKVREILSLREPKSLAICTLLDKPSRREVNVPV 141

tr|G9XZJ2|G9XZJ2_SPIME GQDVLLIEDLIDTGKTLSVVIEQIQAKKPNSLRVVCLADKVAMHSNFNYP 139

.::::::**::*:* *:. : : : : ..*: : : ** : :.

tr|Q7MBB3|Q7MBB3_MYCGA DYVCFEVPHGFLVGFGLDYKDKLRNLPYVAHMVSN-------DKK- 181

tr|G9XX66|G9XX66_SPIME DWYGFDVPPAFLIGFGLDYQERLRNLPYIAIADQEKLATWKWDIKK 195

tr|A9NE16|A9NE16_ACHLI DYIGVTIPKKFVVGYGLDYNEYYRNLPYIGVLKPEVYTK------- 179

sp|P37472|HPRT_BACSU DFVGFEVPDAFVVGYGLDYAERYRNLPYIGVLKPAVYES------- 180

sp|P0A9M2|HPRT_ECOLI EFIGFSIPDEFVVGYGIDYAQRYRHLPYIGKVILLDE--------- 178

tr|G9XZJ2|G9XZJ2_SPIME YDALFTVPNEFIVGYGFDYNDQFRQLPNVYIWRGE----------- 174

. :* *::*:*:** : *:** :

- 1. **Apt. Adenine phosphoribosyltransferase**

Here we used the crystal structure of the Apt protein from *Leishmania donovani* as reference. All substitutions observed in the N-terminal part are necessary for AMP binding (40-42 position).

sp|A9NGG3|APT_ACHLI ----------------------MDLKAHIAAVKDFPKEGILFRDITPLML 28

sp|O34443|APT_BACSU ----------------------MDLKQYVTIVPDYPKEGVQFKDITTLMD 28

sp|P69503|APT_ECOLI --------------MTATAQQLEYLKNSIKSIQDYPKPGILFRDVTSLLE 36

sp|Q7NBS4|APT_MYCGA ----------------MKTELIAQLKKTIITVKDFPKPGILFYDITPILL 34

tr|L5VS29|L5VS29_SPIME ----------------------MDLKKFIVDIPNYPERGVIFRDITPLLN 28

tr|Q27679|Q27679_LEIDO MPFKEVSPNSFLLDDSHALSQLLKKSYRWYSPVFSPRNVPRFADVSSITE 50

. *. * *::.:

sp|A9NGG3|APT_ACHLI DGKAFKYASDQFTEFARSKK--ADLIVGPEARGFIFGCPVAVNLGVGFAP 76

sp|O34443|APT_BACSU KGDVYRYATDQIVEYAKEKQ--IDLVVGPEARGFIIGCPVAYALGVGFAP 76

sp|P69503|APT_ECOLI DPKAYALSIDLLVERYKNAG--ITKVVGTEARGFLFGAPVALGLGVGFVP 84

sp|Q7NBS4|APT_MYCGA DPKLFDQVISVMAEVAKKSN--ADMIASPESRGFLFGVPLANKLKLPFVL 82

tr|L5VS29|L5VS29_SPIME NALAFTTVINQLAAYAVAQK--ATVIIAPEARGFLFGPAVSYATNLRFIP 76

tr|Q27679|Q27679_LEIDO SPETLKAIRDFLVQRYRAMSPAPTHILGFDARGFLFGPMIAVELEIPFVL 100

. . :. : . ::***::* :: : *

sp|A9NGG3|APT_ACHLI VRKPGKLPRASVT-VSYDLEYGSN---SLSLHEDAVKPGQRVVIIDDLLA 122

sp|O34443|APT_BACSU VRKEGKLPREVIK-VDYGLEYGKD---VLTIHKDAIKPGQRVLITDDLLA 122

sp|P69503|APT_ECOLI VRKPGKLPRETIS-ETYDLEYGTD---QLEIHVDAIKPGDKVLVVDDLLA 130

sp|Q7NBS4|APT_MYCGA VRKQNKLPRATFS-ASYDLEYGKNN--VIEIHQDAIKPNSKVMIVDDLLA 129

tr|L5VS29|L5VS29_SPIME VRKVGKLPRPVIN-VKYSYEYAEN---QLEIHAGDLQPSDRVLIVDDVLA 122

tr|Q27679|Q27679_LEIDO MRKADKNAGLLIRSEPYEKEYKEAAPEVMTIRYGSIGKGSRVVLIDDVLA 150

:** .* . . * ** : :: . : ..:*:: **:**

sp|A9NGG3|APT_ACHLI TGGTMQATVELVEKLGGIVVGLAFLIELDDLEGRKLLKNYDVK--TLINY 170

sp|O34443|APT_BACSU TGGTIEATIKLVEELGGVVAGIAFLIELSYLDGRNKLEDYDIL--TLMKY 170

sp|P69503|APT_ECOLI TGGTIEATVKLIRRLGGEVADAAFIINLFDLGGEQRLEKQGITSYSLVPF 180

sp|Q7NBS4|APT_MYCGA TAGTVDAISRLVKQAKSEVVSYSFLIRLKDLGGIDKLDQTKPID-YILEY 178

tr|L5VS29|L5VS29_SPIME SGGTARAMCQLVNQEQATVAGLAFVIDLTYLNGKKDLDSYPIK--TLIEY 170

tr|Q27679|Q27679_LEIDO TGGTALSGLQLVEASDAVVVEMVSILSIPFLKAAEKIHSTANSRYKDIKF 200

:.** : .*:. . *. :: : * . . :.. : :

sp|A9NGG3|APT_ACHLI -------------------------------------

sp|O34443|APT_BACSU -------------------------------------

sp|P69503|APT_ECOLI PGH---------------------------------- 183

sp|Q7NBS4|APT_MYCGA -------------------------------------

tr|L5VS29|L5VS29_SPIME -------------------------------------

tr|Q27679|Q27679_LEIDO ISLLSDDALTEENCGDSKNYTGPRVLSCGDVLAEHPH 237

**5.4 PlsY. Glycerol-3-phosphate acyltransferase**

Amino acids of active center are unknown.

sp|Q8L3A1|PLSY_ACHLA ---MNMQILWIIIAIICSYLIGAIPFGYIIPKLFKGIDIREHGSKNVGST 47

sp|Q45064|PLSY_BACSU --------MLIALLIILAYLIGSIPSGLIVGKLAKGIDIREHGSGNLGAT 42

tr|G9XYX8|G9XYX8_SPIME --MTLYGYLGTAITAIIGYLIGSFSWSIFISKKIYKIDVRDYHSKNAGAT 48

sp|Q7NB93|PLSY_MYCGA MNVAGYISLLVILSLLIGYLFGSIMFADVASMILKR-NVRELGSKNPGTT 49

sp|P60782|PLSY_ECOLI ------MSAIAPGMILIAYLCGSISSAILVCRLCGLPDPRTSGSGNPGAT 44

: .** *:: . . : * * * *:*

sp|Q8L3A1|PLSY_ACHLA NVLRVLGAKYGIPTFLLDCFKGALPIIIIRYMLGMPELFLISDTYDI--- 94

sp|Q45064|PLSY_BACSU NAFRTLGVKAGSVVIAGDILKGTLATALP--------FLMHVDIH----- 79

tr|G9XYX8|G9XYX8_SPIME NTSRVLGKKWGFAIMFLDMLKVTITMFIAFGISCININGVNFGSTS---- 94

sp|Q7NB93|PLSY_MYCGA NSFRVFPKKVAIAIGFFEIIKSVIPFSIILLIYKYGLQPELTKLDPSVIN 99

sp|P60782|PLSY_ECOLI NVLRIGGKGAAVAVLIFDVLKGMLP---VWGAYELGVSP----------- 80

* * . : :* :.

sp|Q8L3A1|PLSY_ACHLA -----SIVFGAAAAIGHIKSIYIGFKGGKAVATGVGAVIAINPIIGLSGI 139

sp|Q45064|PLSY_BACSU -----PLLAGVFAVLGHVFPIFAKFKGGKAVATSGGVLLFYAPLLFITMV 124

tr|G9XYX8|G9XYX8_SPIME -----YYIPAFFVLIGHSYPIYYKFKGGKTVSSFLGLLWMTNPYYFLIAT 139

sp|Q7NB93|PLSY_MYCGA KTYYLTYLAPLAAIFGHMYPVYFKFNGGKAVATTAGFVFVVSPWWFLIIA 149

sp|P60782|PLSY_ECOLI ---FWLGLIAIAACLGHIWPVFFGFKGGKGVATAFGAIAPIGWDLTGVMA 127

: . :** .:: *:*** *:: * :

sp|Q8L3A1|PLSY_ACHLA GLFFIVAFSTKYVSIGSVVASFSVAVMMWIGVL----------------- 172

sp|Q45064|PLSY_BACSU AVFFIFLYLTKFVSLSSMLTGIYTVIYSFF-------------------- 154

tr|G9XYX8|G9XYX8_SPIME VVWWSTIFIWKRVSVSSILAALFTGALCWIPQLSGIDIINFNGDLLQNSH 189

sp|Q7NB93|PLSY_MYCGA LTWWTITLISKYVSLASIVCFIIFLFLPYIPWL-------------DYLW 186

sp|P60782|PLSY_ECOLI GTWLLTVLLSGYSSLGAIVSALIAPFY---------------------VW 156

: *:.::: :

sp|Q8L3A1|PLSY_ACHLA -IKEIWIPVPNLTISYESQIINLVAISLIVLLIIYMHKKNFIRLMNGTEN 221

sp|Q45064|PLSY_BACSU -VHDTYL---------------LIVVTLLTIFVIYRHRANIKRIINKTEP 188

tr|G9XYX8|G9XYX8_SPIME LVWVNYLHYVNYDNYYDSLALINIIITLSAIFLILKHHQNITRLLKGTEK 239

sp|Q7NB93|PLSY_MYCGA WFSLDKITFLTYQSDWYIIVFFAIANTILSTIIIWKHRGNIVRLINKQEN 236

sp|P60782|PLSY_ECOLI WFKP----------------QFTFPVSMLSCLILLRHHDNIQRLWRRQET 190

. . :: ::: *: *: *: . *

sp|Q8L3A1|PLSY_ACHLA KIGQKKIQNITK------------ 233

sp|Q45064|PLSY_BACSU KVKWL------------------- 193

tr|G9XYX8|G9XYX8_SPIME PYDFKGKSDLENGNLSKHNKIKKH 263

sp|Q7NB93|PLSY_MYCGA KITKKV------------------ 242

sp|P60782|PLSY_ECOLI KIWTKFKRKREKDPE--------- 205

- 1. **PlsX. Phosphate acyltransferase**

There are no significant amino acid substitutions in the active site.

sp|P71018|PLSX_BACSU ---MRIAVDAMGGDHAPKAVIDGVIKGIEAFDDLHITLVGDKTTIESHLT 47

sp|Q82ZE8|PLSX_ENTFA ---MKIAVDAMGGDNAPQAIVEGVMLAKQDFPDIEFQLYGKEAEIKKYIT 47

sp|P27247|PLSX_ECOLI MTRLTLALDVMGGDFGPSVTVPAALQALNSNSQLTLLLVGNSDAITPLLA 50

sp|A9NES3|PLSX_ACHLI --MIKLAIDGMGGDNAPKEIVEGSILALKQFDDIELTIFGDVDKMKPYLI 48

sp|Q7NAZ1|PLSX_MYCGA --MFRIAVDCMGFENSVSEAVKAVIKYAKKHKDLSFVLVGDENQIRPLVE 48

tr|G9XXK9|G9XXK9_SPIME --MSKIAIDMMGTDLGIKPIIDALKVFMKKYDDVTFVLIGDEKELKSATA 48

:*:* ** : . . : . : :: : : *. :

sp|P71018|PLSX_BACSU ----TTSDRITVLHADEVIEPTDEPV--RAVRRKKNSSMVLMAQEVAENR 91

sp|Q82ZE8|PLSX_ENTFA ----DEKN-ITIIHTDEKIASDDEPV--KAIRRKKTASMVLAAQAVKNGE 90

sp|P27247|PLSX_ECOLI KADFEQRSRLQIIPAQSVIASDARPS--QAIRASRGSSMRVALELVKEGR 98

sp|A9NES3|PLSX_ACHLI -----EHPRLKVVHTPKYFEMGVKDIGRHTLRDDKDTSMLMAINHVKEGL 93

sp|Q7NAZ1|PLSX_MYCGA NK-KYLN--YRIVHTTNEIGMSDSVL---TAYRKKDSSMYLTIELLKNNE 92

tr|G9XXK9|G9XXK9_SPIME AA-KLDNTKYEIFATTEVIAMMEGMM---EVRRKPNSSMVRGTELLRDQK 94

:. : . : . :** : : :

sp|P71018|PLSX_BACSU ADACISAGNTGALMTAGLFIVGRIKGIDRPALAPTLPTVS---GDGFLLL 138

sp|Q82ZE8|PLSX_ENTFA ADAIFSAGNTGALLAAGLFIVGRIKNVERPGLMSTLPVMGE-PDKGFDML 139

sp|P27247|PLSX_ECOLI AQACVSAGNTGALMGLAKLLLKPLEGIERPALVTVLPHQQ---KGKTVVL 145

sp|A9NES3|PLSX_ACHLI ADGVVSSGPTQALIFASFFMIRPMKEMKRVAIAPMVPTVI---GKPTILL 140

sp|Q7NAZ1|PLSX_MYCGA VDTIISAGSSSAYVALTYNLIGKIHHKIKVGFMSYVPTVTK---RGFWFL 139

tr|G9XXK9|G9XXK9_SPIME VDALVSGGSTAAFLAACHFIVGEISGVSRPAFMPFIPTVNKG--KSVLML 142

.: .*.* : * : :: : : .: . :* .*

sp|P71018|PLSX_BACSU DVGANVDAKPEHLVQYAIMGSVYSQQVRGVTSPRVGLLNVGTEDKKGNEL 188

sp|Q82ZE8|PLSX_ENTFA DLGANADNKPEHLVQYAVLGSFYAEKVRNVQNPRVGLLNNGTEETKGSEL 189

sp|P27247|PLSX_ECOLI DLGANVDCDSTMLVQFAIMGSVLAEEVVEIPNPRVALLNIGEEEVKGLDS 195

sp|A9NES3|PLSX_ACHLI DAGGNIDAKAEHLLDFAIFSTIALKEVYGVKSPKVGLINIGTEPGKGRDI 190

sp|Q7NAZ1|PLSX_MYCGA DVGANKEYLGEELYYLGKMANTFITSVFN-YQPRLGVLNIGAEKNKGFEY 188

tr|G9XXK9|G9XXK9_SPIME DVGANLENDAQDLVNFAIMASVYAQEIMHNPKPSVAILNIGEEASKGKDY 192

* *.* : * . :.. .: .* :.::* * * ** :

sp|P71018|PLSX_BACSU TKQTFQILKETANINFIGNVEARDLLDDVADVVVTDGFTGNVTLKTLEGS 238

sp|Q82ZE8|PLSX_ENTFA TKKAFELLAADETINFVGNVEARELLNGVADVVVTDGFTGNAVLKSIEGT 239

sp|P27247|PLSX_ECOLI IRDASAVLKTIPSINYIGYLEANELLTGKTDVLVCDGFTGNVTLKTMEGV 245

sp|A9NES3|PLSX_ACHLI DKETFELLSKHPLIDFYGNLEPKEILTSDAQILLSDGFTANIVMKTMEGT 240

sp|Q7NAZ1|PLSX_MYCGA HQVVYNLLENDKTVDFLGFIEPRGLIKGECDLLVSDGYSGNLVLKSLEGA 238

tr|G9XXK9|G9XXK9_SPIME HKEAYKLLANNKKIFFKGNIEPRDITSDVVDIVVTDGFTGNIALKTLEGM 242

: . :* : : * :*.. : . :::: **::.* .:*::**

sp|P71018|PLSX_BACSU ALSIFKMMRDVMTST-LTSKLAAAV--LKPKLKEMKMKMEYSNYGGASLF 285

sp|Q82ZE8|PLSX_ENTFA AMNMMSLLKTAILSEGVKGKMGALL--LKNALHGMKDEMDYSKHGGAVLF 287

sp|P27247|PLSX_ECOLI VRMFLSLLKSQGEGKKRSWWLLLLKRWLQKSLTRRFSHLNPDQYNGACLL 295

sp|A9NES3|PLSX_ACHLI ASALGKILKREIKAS-FWGKLAAVL-FLKKPLKRFKQSMSADEVGGALIA 288

sp|Q7NAZ1|PLSX_MYCGA LKSVGKILKKNYKIN---PLGALFS---ANVIYQITKTFDYKNNAGAVVL 282

tr|G9XXK9|G9XXK9_SPIME AKNLMGVIKHELTKNFYRKLKAFSL---RKAFRGVRETFDYRNNAAALVL 289

. ::: : :. : .* :

sp|P71018|PLSX_BACSU GLKAPVIKAHGSSDSNAVFHAIRQAREMVSQNVAALIQEEVK-------- 327

sp|Q82ZE8|PLSX_ENTFA GLKAPVIKTHGATGPDAVRYTIRQIHTMLETQVVPQLVEYY--------- 328

sp|P27247|PLSX_ECOLI GLRGTVIKSHGAANQRAFAVAIEQAVQAVQRQVPQRIAARLESVYPAGFE 345

sp|A9NES3|PLSX_ACHLI GLDKVVVKAHGSSEAYAFMNAIRQAKTMVSHDVIGKVKNVLR-------- 330

sp|Q7NAZ1|PLSX_MYCGA GLNKLVLKTHGSADAKQFYSTIRLAHESLLNNLIEKITKECSTFLN---- 328

tr|G9XXK9|G9XXK9_SPIME GLKAIAVKTHGSSDAKSWISTLELTRTAVVNDFVQKMTVKLLKGD----- 334

** .:*:**:: ::. : :. :

sp|P71018|PLSX_BACSU ---EEKTDE-- 333

sp|Q82ZE8|PLSX_ENTFA ---EGKAE--- 333

sp|P27247|PLSX_ECOLI LLDGGKSGTLR 356

sp|A9NES3|PLSX_ACHLI ----GQDE--- 334

sp|Q7NAZ1|PLSX_MYCGA -----------

tr|G9XXK9|G9XXK9_SPIME -----------

- 1. **Fba. Fructose-bisphosphate aldolase**

Here we used the crystal structure of the Fba protein from *Bacillus anthracis* as our standard. We found (relative *B.* *anthracis*) V50A substitution in Q7NAJ0_MYCGA and G9XY54_SPIME but not in *A. laidlawii*. This position is located in substrate binding site; it may hypothetically affect enzyme activity.

tr|A9NE26|A9NE26_ACHLI -------MLVSAKEMLQKAKAEGYAVAQININNLEWIKAVLSTVEELKSP 43

tr|G9XY54|G9XY54_SPIME MGQKYHARLVNASELIKKAHQNKYAVGHFNINNLEWTKALLEAAQATKTP 50

sp|P13243|ALF_BACSU ------MPLVSMTEMLNTAKEKGYAVGQFNLNNLEFTQAILQAAEEEKSP 44

tr|Q81JW4|Q81JW4_BACAN ------MPLVSMKEMLNKALEGKYAVGQFNMNNLEWTQAILAAAEEEKSP 44

**. . :::.* **:.::* ****: :*:* :.: ::*

tr|Q7NAJ0|Q7NAJ0_MYCGA VIVAASEGAVKYMGGYQAVYHIVNDAINNLNITVPVALHLDHG-TYEGVF 94

tr|A9NE26|A9NE26_ACHLI VILGVSEGAAKYMGGYENVMAMVSTLDKAMNISVPVAVHLDHG-TYEGAF 92

tr|G9XY54|G9XY54_SPIME IILGASEGAIKYMGGYNLVVAMVNALLDSLDITVPVALHLDHGQSVESCK 100

sp|P13243|ALF_BACSU VILGVSEGAGRYMGGFKTVVAMVKALMEEYKVTVPVAIHLDHGSSFESCA 94

tr|Q81JW4|Q81JW4_BACAN VILGVSEGAARHMTGFKTVVAMVKALIEEMNITVPVAIHLDHGSSFEKCK 94

:*:..**** ::* *:: * :*. . .::****:***** : *

tr|Q7NAJ0|Q7NAJ0_MYCGA KALEAGFSSVMFDGSHLPFAENYEKSIKVIEAAKKYNASVELEVGTIGGE 144

tr|A9NE26|A9NE26_ACHLI KAIRAGFTSVMFDGSHYPFEENLKKTQEVVAVAHAVGVSVEAEVGSIGGE 142

tr|G9XY54|G9XY54_SPIME MAIDAGFSSVMYDGSHHPFAENLKNTKEVVAYAKTNAVSVEVEIGTIGGE 150

sp|P13243|ALF_BACSU KAIHAGFTSVMIDASHHPFEENVATTAKVVELAHFHGVSVEAELGTVGGQ 144

tr|Q81JW4|Q81JW4_BACAN EAIDAGFTSVMIDASHHPFEENVETTKKVVEYAHARNVSVEAELGTVGGQ 144

*: ***:*** *.** ** ** .: :*: *: .*** *:*::**:

tr|Q7NAJ0|Q7NAJ0_MYCGA EDGVVGNGEL-ANPQECKKMKD-LGCDMLAAGIGNIHGIYPPTWKSLNFE 192

tr|A9NE26|A9NE26_ACHLI EDGVVGTGEL-ADPEECRIIAA-TGVDLFAAGIGNIHGKYPANWKGLDFG 190

tr|G9XY54|G9XY54_SPIME EDGVVGAGEI-GDPKEAAEMVA-TGIDFLAAGIGNIHGPYPTGWPGLNFQ 198

sp|P13243|ALF_BACSU EDDVIAEGVIYADPKECQELVERTGIDCLAPALGSVHGPYK-GEPNLGFK 193

tr|Q81JW4|Q81JW4_BACAN EDDVIAEGVIYADPAECKHLVEATGIDCLAPALGSVHGPYK-GEPNLGFA 193

**.*:. * : .:* *. : * * :*..:*.:** * .*.*

tr|Q7NAJ0|Q7NAJ0_MYCGA VLSELKKASD-ASLVLHGGSGIPNEQVKKAISLGVTKVNVNTECQLAFAS 241

tr|A9NE26|A9NE26_ACHLI VLKTVSEVTNGVPLVLHGGTGIPADQITKAISLGIAKINVNTELQLAFAA 240

tr|G9XY54|G9XY54_SPIME ALEDIQAAAK-IGMVLHGGSGIPQEQVKKAISLGISKINVNTELQIAFAV 247

sp|P13243|ALF_BACSU EMEEIGKSTG-LPLVLHGGTGIPTADIKKSISLGTAKINVNTENQISSAK 242

tr|Q81JW4|Q81JW4_BACAN EMEQVRDFTG-VPLVLHGGTGIPTADIEKAISLGTSKINVNTENQIEFTK 242

:. : : :*****:*** :: *:**** :*:***** *: :

tr|Q7NAJ0|Q7NAJ0_MYCGA ATRKYILEEKDLDQHKKGYDPRKLLKPGFEAIKATCIEKIKLFGSENKA 290

tr|A9NE26|A9NE26_ACHLI ATRKYIEEGKDLES--KGFDPRKLLNPGYVAMKQVIKDKLTMFGSVNKA 287

tr|G9XY54|G9XY54_SPIME ATRKYIEEGKDKPENGKGFDPRKLLKPGYEAIKTTFDELTSWFGCKGKA 296

sp|P13243|ALF_BACSU AVRETLA--AKPDE----YDPRKYLGPAREAIKETVIGKMREFGSSNQA 285

tr|Q81JW4|Q81JW4_BACAN AVREVLN--KDQEV----YDPRKFIGPGRDAIKATVIGKIREFGSNGKA 285

*.*: : . :**** : *. *:* . **. .:*

- 1. **PrsA. Ribose-phosphate pyrophosphokinase**

We found (relative to *E. coli*) two substitutions in ATP binding loop: R107A in Q7NAR0_MYCGA, S108G in Q7NAR0_MYCGA and G9XZ29_SPIME; two substitutions in ATP binding sites: S310P in A9NE81_ACHLI and F315Y in Q7NAR0_MYCGA and G9XZ29_SPIME; and two substitutions in the phosphoribosylpyrophosphate binding loop: M222I and L218M in A9NE81_ACHLI protein.

tr|A9NE81|A9NE81_ACHLI MT--IDEKKAKLFTLSANKPLAEKIAKSAGIPLSNVEVIRFADGEITVNI 48

sp|P14193|KPRS_BACSU MSNQYGDKNLKIFSLNSNPELAKEIADIVGVQLGKCSVTRFSDGEVQINI 50

sp|P0A717|KPRS_ECOLI MP------DMKLFAGNATPELAQRIANRLYTSLGDAAVGRFSDGEVSVQI 44

tr|G9XZ29|G9XZ29_SPIME ME----EKSFSIYGLSAGIKLADEICKILGVTRQEMETVRFADGEILVRA 46

tr|Q7NAR0|Q7NAR0_MYCGA MH---VKSNHIIFGLSAAKKLTDSICRRLSMKPGEIVIQKFADDEIFVRP 47

* . :: .: *:. *. . :*:*.*: :.

tr|A9NE81|A9NE81_ACHLI EESVRGNHVFVIQPTSEPANDHLMEVLVLTDALKRASAASITIIMPYFGY 98

sp|P14193|KPRS_BACSU EESIRGCDCYIIQSTSDPVNEHIMELLIMVDALKRASAKTINIVIPYYGY 100

sp|P0A717|KPRS_ECOLI NENVRGGDIFIIQSTCAPTNDNLMELVVMVDALRRASAGRITAVIPYFGY 94

tr|G9XZ29|G9XZ29_SPIME MNSVRGKDVYIIQSTSWPVNENLMELLIAIDALKRGSAQSINVIIPYFGY 96

tr|Q7NAR0|Q7NAR0_MYCGA DCTLRNKDVVFIQSTNSPVNDNLMELLIAIDSAKRASAKSITALIPYYGY 97

.:*. . .**.* *.*:::**::: *: :*.** *. ::**:**

tr|A9NE81|A9NE81_ACHLI SRQDRKVKS-RQPITAKLVANLLTVAGVDRVVSIDLHAAQIQGFFDIPID 147

sp|P14193|KPRS_BACSU ARQDRKARS-REPITAKLFANLLETAGATRVIALDLHAPQIQGFFDIPID 149

sp|P0A717|KPRS_ECOLI ARQDRRVRSARVPITAKVVADFLSSVGVDRVLTVDLHAEQIQGFFDVPVD 144

tr|G9XZ29|G9XZ29_SPIME ARQDRKARG-RQPITCKLVANMLTTAGATRVMTVDLHSPQSMGFFDVPVD 145

tr|Q7NAR0|Q7NAR0_MYCGA ARQDRKSAG-REPITSKLVADLLTKAGVTRATLTDIHSDQTQGFFDIPVD 146

:****: . * ***.*:.*::* .*. *. *:*: * ****:*:*

tr|A9NE81|A9NE81_ACHLI NFPAAPTLASYFRRKKLEN-----VVVVSPDHGGVTRARVFASFFNAP-- 190

sp|P14193|KPRS_BACSU HLMGVPILGEYFEGKNLED-----IVIVSPDHGGVTRARKLADRLKAP-- 192

sp|P0A717|KPRS_ECOLI NVFGSPILLEDMLQLNLDN-----PIVVSPDIGGVVRARAIAKLLNDTD- 188

tr|G9XZ29|G9XZ29_SPIME DLRSTQELVRTIVRKIEEDHLKEEVTIVSPDHGGLVRARDVANRLGNLAG 195

tr|Q7NAR0|Q7NAR0_MYCGA ILKASFVLLSHVLVDNKID----DLVVVSPDYGGVKRARKIAESINVP-- 190

. . * . : :**** **: *** .*. :

tr|A9NE81|A9NE81_ACHLI -LAIIDKRRPEPNKAEVMNIIGDVKGATCIMIDDIIDTGGTLMAGANALK 239

sp|P14193|KPRS_BACSU -IAIIDKRRPRPNVAEVMNIVGNIEGKTAILIDDIIDTAGTITLAANALV 241

sp|P0A717|KPRS_ECOLI -MAIIDKRRPRANVSQVMHIIGDVAGRDCVLVDDMIDTGGTLCKAAEALK 237

tr|G9XZ29|G9XZ29_SPIME NIAVIDKRRPKPNVSEVQFILGDVKDRICFIVDDMIDTAGTICNAAKALK 245

tr|Q7NAR0|Q7NAR0_MYCGA -LAIIDKRRPKPNVAESINILGEVKNKCCILVDDMIDTGGTIISAAKLLK 239

:*:******..* :: *:*:: . ..::**:***.**: .*: *

tr|A9NE81|A9NE81_ACHLI EAGAKEVYAAATHGVLTSNATER----LQNSVINEIVITDTIYLDPA--K 283

sp|P14193|KPRS_BACSU ENGAKEVYACCTHPVLSGPAVER----INNSTIKELVVTNSIKLPEE--K 285

sp|P0A717|KPRS_ECOLI ERGAKRVFAYATHPIFSGNAANN----LRNSVIDEVVVCDTIPLSDEI-K 282

tr|G9XZ29|G9XZ29_SPIME QHGAKAVYLLACHGVFSPPAKERLTELINDGTVKKVIVTNTIDINQD--R 293

tr|Q7NAR0|Q7NAR0_MYCGA KQKAKTVLVMATHGLFNKNAIELFDECYKKKEINKVYIADTIDQTEKNLE 289

: ** * . * ::. * : .. :.:: : ::* .

tr|A9NE81|A9NE81_ACHLI NQPKLKQLSIGALLGEAIIHILQDEPISQIFNRIQEDQ------------ 321

sp|P14193|KPRS_BACSU KIERFKQLSVGPLLAEAIIRVHEQQSVSYLFS------------------ 317

sp|P0A717|KPRS_ECOLI SLPNVRTLTLSGMLAEAIRRISNEESISAMFEH----------------- 315

tr|G9XZ29|G9XZ29_SPIME LFDGLEIISIADLISQMIKAMIEKRSLSDVYSKCNEQIK-----KIIEKI 338

tr|Q7NAR0|Q7NAR0_MYCGA KKPQFKVVKLDHFYSLVLDKYINGGSISDVYTIYQEWVKSPNLKKILKEI 339

.. :.: : . : : .:* ::

tr|A9NE81|A9NE81_ACHLI --

sp|P14193|KPRS_BACSU --

sp|P0A717|KPRS_ECOLI --

tr|G9XZ29|G9XZ29_SPIME KK 340

tr|Q7NAR0|Q7NAR0_MYCGA E- 340

- 1. **TktA. Transketolase**

There is one substitution in the substrate binding site: S384A (relative to *E. coli*) in G9Y039_SPIME protein.

Two transketolase coding genes were annotated for *M. gallisepticum* and *S. melliferum.* We believe the annotation for Q7NAR4_MYCGA incorrect because all the amino acids in the active site are replaced.

sp|P27302|TKT1_ECOLI ------------MSSRKELANAIRALSMDAVQKAKSGHPGAPMGMADIAE 38

sp|P33570|TKT2_ECOLI -------------MSRKDLANAIRALSMDAVQKANSGHPGAPMGMADIAE 37

sp|P45694|TKT_BACSU -----------MDTIEKKSVATIRTLSIDAIEKANSGHPGMPMGAAPMAY 39

tr|A9NEC3|A9NEC3_ACHLI ------------MDINKLSIDTIRFLGIDAINKANSGHPGIVIGAAPMAH 38

tr|G9XYY6|G9XYY6_SPIME ---------------MKKSIDTIKMLGIEAINKANSGHPGIVLGAAPMAY 35

tr|G9Y039|G9Y039_SPIME -------MKSTNNNIETKSLSNLRILGLDPIIYNKTGHPGIVLSAAPLMQ 43

tr|Q7NC51|Q7NC51_MYCGA ------------MNIQQQTINTIRVLGIEMINNAKSGHPGMVMSAAPMMY 38

tr|Q7NAR4|Q7NAR4_MYCGA MVIKLIYQNKPFPKNSNSIVNSFRFLMFDTVNNNFVGSAAYGFGAASYLY 50

:: * :: : * .. :. *

sp|P27302|TKT1_ECOLI VLWRDFLKHNPQNPSWADRDRFVLSNGHGSMLIYSLLHLTGYD-LPMEEL 87

sp|P33570|TKT2_ECOLI VLWNDFLKHNPTDPTWYDRDRFILSNGHASMLLYSLLHLTGYD-LPLEEL 86

sp|P45694|TKT_BACSU TLWTKFMNVSPANPGWFNRDRFVLSAGHGSALLYSMLHLSGFD-LSIEDL 88

tr|A9NEC3|A9NEC3_ACHLI TLFTKHINIYPKMSRWINRDRFILSAGHGSMLLYALNHLSGYK-VSIDDL 87

tr|G9XYY6|G9XYY6_SPIME TLFTRHLVVNPQVDKWTNRDRFVLAAGHGSALLYSLLHLSGFD-LNIYDL 84

tr|G9Y039|G9Y039_SPIME AIYLDNLIANPAVPDWINRDRFVLSPGHASTLQYAILHLAGYN-LTIDDL 92

tr|Q7NC51|Q7NC51_MYCGA ALFHDHLNYDVSDPNYLNRDRFILSAGHGSALLYATMYVAGYKTLSTKDL 88

tr|Q7NAR4|Q7NAR4_MYCGA VLFRNYFVMDLDNLDANYNDKLVVSKQLGGSNIRACFYLLKHPDIKIEDL 100

.:: : .*::::: .. : :: . : :*

sp|P27302|TKT1_ECOLI KNFRQLHSKTPGHPEVGYTAGVETTTGPLGQGIANAVGMAIAEKTLAAQF 137

sp|P33570|TKT2_ECOLI KNFRQLHSKTPGHPEIGYTPGVETTTGPLGQGLANAVGLAIAERTLAAQF 136

sp|P45694|TKT_BACSU KGFRQWGSKTPGHPEFGHTAGVDATTGPLGQGIAMAVGMAIAERHLAETY 138

tr|A9NEC3|A9NEC3_ACHLI KNFRNYPGNTPGHPEYGHTDGVETTSGPLGQGISNAVGMAIAEKHLAARF 137

tr|G9XYY6|G9XYY6_SPIME RNFRQVDSITPGHPESHLTPGVDVTTGPLGQGLAAAVGLALAETHLAAKY 134

tr|G9Y039|G9Y039_SPIME KNYRHINSKTPAHPEYGVTPGVDNSSGPLGQGVGYGVGMALAEQHLAAKF 142

tr|Q7NC51|Q7NC51_MYCGA KNFRKFSSKTPGHPESTMLAGVDFGTGPLGQGAATSVGFAIAEANLSARF 138

tr|Q7NAR4|Q7NAR4_MYCGA MSFSHEKQIRNLYDFSTYQPGYN---------LAYAVGLAIDAK----LV 137

.: : : * : . .**:*:

sp|P27302|TKT1_ECOLI NRPGHDIVDHYTYAFMGDGCMMEGISHEVCSLAGTLKLGKLIAFYDDNGI 187

sp|P33570|TKT2_ECOLI NQPDHEIVDHFTYVFMGDGCLMEGISHEVCSLAGTLGLGKLIGFYDHNGI 186

sp|P45694|TKT_BACSU NRDSFNVVDHYTYSICGDGDLMEGISSEAASLAGHLQLGRLIVLYDSNDI 188

tr|A9NEC3|A9NEC3_ACHLI NEPNFDLISHYTYVLVGDGDLQEGVALEAISLAGHLGLGKLIVLYDSNDI 187

tr|G9XYY6|G9XYY6_SPIME NTKKYPLYDHYTYVLCGDGDLQEGVTQEAISLAGHWKLNKLIVLFDSNDV 184

tr|G9Y039|G9Y039_SPIME NKPDYKIIDHYTYVLCSDGDLQEGGAIEAIQLAGVWKLNKLIMLYDSNDC 192

tr|Q7NC51|Q7NC51_MYCGA DK----IINHYTYCLIGDGDLQEGVCQEALAVAGRYKLNKLIWLYDSNDV 184

tr|Q7NAR4|Q7NAR4_MYCGA NQKSHDTITNKIYCIVSAADLNSSYGLAALKTAANQELNNLIIIYDNNHF 187

: : * : . . : .. . *. *..** ::* *

sp|P27302|TKT1_ECOLI SIDGHVEGWFTDDTAMRFEAYGWHVIRDIDG-HDAASIKRAVEEARAVTD 236

sp|P33570|TKT2_ECOLI SIDGETEGWFTDDTAKRFEAYHWHVIHEIDG-HDPQAVKEAILEAQSVKD 235

sp|P45694|TKT_BACSU SLDGDLDRSFSENVKQRFEAMNWEVLYVEDG-NNIEELTAAIEKARQNEK 237

tr|A9NEC3|A9NEC3_ACHLI QLDGETNLAISEDVKKKFEAQNWHYTKVEDG-EDLDQINRAILRAKKIVD 236

tr|G9XYY6|G9XYY6_SPIME QLDNMVNVAQSENIADRFKAANWNYLFVKDG-NDIEAIDQAIIKAKK-SN 232

tr|G9Y039|G9Y039_SPIME QLDTKCDAVLKIDYQRFFEAQNWNYIRIENADEDLPAIQKAIEQAQK-SD 241

tr|Q7NC51|Q7NC51_MYCGA QLDGRVENSTNFDVEMLLKSYRWNYILIKDG-NDYQAISNAIAQAKK-SD 232

tr|Q7NAR4|Q7NAR4_MYCGA EERGENQDYLVTDFSSLVKDMGFKYINVFNG-NNIEKLDAGFHYAVN-SK 235

. : : .: :. :. .: : .. * .

sp|P27302|TKT1_ECOLI KPSLLMCKTIIGFGSPNKAGTHDSHGAPLGDAEIALTREQLGWKYAP-FE 285

sp|P33570|TKT2_ECOLI KPSLIICRTVIGFGSPNKAGKEEAHGAPLGEEEVALARQKLGWHHPP-FE 284

sp|P45694|TKT_BACSU KPTLIEVKTTIGFGSPNRAGTSGVHGAPLGKEESKLTKEAYAWTYEEDFY 287

tr|A9NEC3|A9NEC3_ACHLI KPTIIEVKTIIGRG-TTSEGTSKVHGSPLGEAEREKLAQKSGYNYKP-FE 284

tr|G9XYY6|G9XYY6_SPIME KPTLIEVKTIIGDG-ATKQGTPAVHGAPLG-SDIETVRKMLEWNYKP-FE 279

tr|G9Y039|G9Y039_SPIME KPTLIECKTIIGYG-HPKQGSP-MHSSPFTPEEMDQVKAFYDFNHPQ-FY 288

tr|Q7NC51|Q7NC51_MYCGA KPTFIEVKTKLGFA-SSVENTNKAHGSPFSDEEIKNIKAKFDYKNKP-FT 280

tr|Q7NAR4|Q7NAR4_MYCGA KPVFIDINTIVGHG-YDSAGTKEVIKSSLTQDQLDQLTKRFEYTGEE-FM 283

** :: .* :* . .. :.: : : *

sp|P27302|TKT1_ECOLI IPSEIYAQWD--AKEAGQAKESAWNEKFAAYAKAYPQEAAEFTRRMKGEM 333

sp|P33570|TKT2_ECOLI IPKEIYHAWD--AREKGEKAQQSWNEKFAAYKKAHPQLAEEFTRRMSGGL 332

sp|P45694|TKT_BACSU VPSEVYEHFAVAVKESGEKKEQEWNAQFAKYKEVYPELAEQLELAIKGEL 337

tr|A9NEC3|A9NEC3_ACHLI VDGEVYSFYKSKVFNKGKRVYNKWVKMLDAYKAQFPEKHDLFNQFLSGNM 334

tr|G9XYY6|G9XYY6_SPIME VPSEVYKDFEVNVKQRGIKKYQQWLKMYQGLYKENPALAKELDDAVYGNF 329

tr|G9Y039|G9Y039_SPIME VDNDVKKHWQDTFAKRGAIKYEEWKKKITAYKAAFPKEYEELFATPSVDL 338

tr|Q7NC51|Q7NC51_MYCGA LSTEVKNHFES-AISRGLKAAEEFNQRLAKAK---PELKEKFISQIKEEK 326

tr|Q7NAR4|Q7NAR4_MYCGA VLYETANDLYPNIKERVNKLKELIADQVDQLKQNNLVIPNYLSAFKDWAL 333

: : . . :

sp|P27302|TKT1_ECOLI PSDFDAKAKEFIAKLQANPAKIASRKASQNAIEAFGPLLP-EFLGGSADL 382

sp|P33570|TKT2_ECOLI PKDWEKTTQKYINELQANPAKIATRKASQNTLNAYGPMLP-ELLGGSADL 381

sp|P45694|TKT_BACSU PKDWDQEVPVYE-----KGSSLASRASSGEVLNGLAKKIP-FFVGGSADL 381

tr|A9NEC3|A9NEC3_ACHLI QIDLSSLVYNAGSK-------EATRNVLGKALTLASHQNL-NIVGGSADL 376

tr|G9XYY6|G9XYY6_SPIME NFNPQDFRDLKPIKP------QATRISSGAILDRLSNIIP-GLIGGSADL 372

tr|G9Y039|G9Y039_SPIME KAFAELLTTDKDKK-------AGTRIIMGDVFKYYQQKCLNNMFGGSADL 381

tr|Q7NC51|Q7NC51_MYCGA INFPKSLINEFNVDQY-----DSTRNLFGKVFKELTKIND-NILVVNCDL 370

tr|Q7NAR4|Q7NAR4_MYCGA SEDQ-----------------IDANQSLAKLKADLIRCQSNSVLLNVSDH 366

:. .. .*

sp|P27302|TKT1_ECOLI APSN-LTLWSGSKAINEDAAGNYIHYGVREFGMTAIANGISLHGGFLPYT 431

sp|P33570|TKT2_ECOLI APSN-LTIWKGSVSLKEDPAGNYIHYGVREFGMTAIANGIAHHGGFVPYT 430

sp|P45694|TKT_BACSU AGSNKTTIKNAGDFTAVDYSGKNFWFGVREFAMGAALNGMALHGGLRVFG 431

tr|A9NEC3|A9NEC3_ACHLI TSST-KAKGNDGHFSRDNALARNINFGVREHAMGAVVNGMTLHGGLKSFT 425

tr|G9XYY6|G9XYY6_SPIME SGST-KAKGADGVYSAENRLGRNLAYGVREFAMAAINNGICLHRGLMPFA 421

tr|G9Y039|G9Y039_SPIME GTAT-KIIGYNGSWTTSTPQNNNVHFGVREFAAGTISIGVELHQGLKGFN 430

tr|Q7NC51|Q7NC51_MYCGA SSSTKVVTHNNARFDHGHYQNQFVDVGVREFLAGCIVNGVVAHKGLKAVS 420

tr|Q7NAR4|Q7NAR4_MYCGA DDEFEREVEAID-------PSRIVLLGNWIELAIIIANAISDNKIHLPVI 409

. . * .: :

sp|P27302|TKT1_ECOLI STFLMFVEYARNAVRMAALMKQRQVMVYTHDSIGLGEDGPTHQPVEQVAS 481

sp|P33570|TKT2_ECOLI ATFLMFVEYARNAARMAALMKARQIMVYTHDSIGLGEDGPTHQAVEQLAS 480

sp|P45694|TKT_BACSU GTFFVFSDYLRPAIRLAALMGLPVTYVFTHDSIAVGEDGPTHEPVEQLAS 481

tr|A9NEC3|A9NEC3_ACHLI GAFFVFSDYMKPPIRLAAMMKIPSVFIFSHDSVAVGEDGPTHEPIEQLIG 475

tr|G9XYY6|G9XYY6_SPIME SGFFVFADYMKPAIRLSSLMEIPAIYILSHDSIAVGEDGPTHQPIEQLAM 471

tr|G9Y039|G9Y039_SPIME STFLIFADYMKPCVRMACIQNLPVIFAFSHDSIGVGFDGKSHQPVEQLAM 480

tr|Q7NC51|Q7NC51_MYCGA STFMAFSDYNKPALRLGAINKLSSLYVYSHDSFNVGEDGPTHQPIEQLSA 470

tr|Q7NAR4|Q7NAR4_MYCGA NLDINLIDKTIQNLKLEEKKDQ---ILYLVDNCKELSRKYVNKVIDLISR 456

: : : :: *. :: :: :

sp|P27302|TKT1_ECOLI LRVTPNMSTWRPCDQVESAVAWKYGVERQDGPTALILSRQNLA-QQERT- 529

sp|P33570|TKT2_ECOLI LRLTPNFSTWRPCDQVEAAVGWKLAVERHNGPTALILSRQNLA-QVERT- 528

sp|P45694|TKT_BACSU LRAMPNLSLIRPADGNETAAAWKLAVQSTDHPTALVLTRQNLP-TIDQTS 530

tr|A9NEC3|A9NEC3_ACHLI LRSIPDLNVIRPADANETKAAVEIAFESKDTATAIITSRQNVL-NLEHT- 523

tr|G9XYY6|G9XYY6_SPIME LRSQPNLNVFRPADFNETLGAYHMALQSKHTPSVILITRQDLP-ELEHS- 519

tr|G9Y039|G9Y039_SPIME LRNCPNLNVFRPADIKEAIGCLKASVEAKSTPSALILSRQDTPYQLAET- 529

tr|Q7NC51|Q7NC51_MYCGA LRLIPDVLVYRPANFYELYIALKTAFDKKNHKPVVISTSRSEFDLTKVN- 519

tr|Q7NAR4|Q7NAR4_MYCGA EK---DGIVLQPGTNEELKYSLCNFKAKNKDKTYIILPSSDDLISIQAD- 502

: : :* * . :: . .

sp|P27302|TKT1_ECOLI EEQLANIARGGYVLKDCAG--QPELIFIATGSEVELAVAAYEKLT-AEGV 576

sp|P33570|TKT2_ECOLI PDQVKEIARGGYVLKDSGG--KPDIILIATGSEMEITLQAAEKLA-GEGR 575

sp|P45694|TKT_BACSU EEALAGVEKGAYVVSKSKNE-TPDALLIASGSEVGLAIEAQAELA-KENI 578

tr|A9NEC3|A9NEC3_ACHLI --SKEGVLKGAYIVSKEEN--KLDGILLASGSEVGLAYEAKQILK-EKGL 568

tr|G9XYY6|G9XYY6_SPIME --NVELVKKGAYQVYGQED--NNHVVLLATGSEVSMAIAVAKKLEHDKKI 565

tr|G9Y039|G9Y039_SPIME --CWEQTLKGGYIVVAEDETKPLEAIIIATGTEVAPAIVAAKTIK----K 573

tr|Q7NC51|Q7NC51_MYCGA ---PKTFDEGYYYLNKTKS---PKLRLIATGSDAGTALKLAKIFK-QEGI 562

tr|Q7NAR4|Q7NAR4_MYCGA -----KINFGGYELYGDSY---ATVNIVTAGSDLIKALDLRQQLL-ENKI 543

* * : ::::*:: : :

sp|P27302|TKT1_ECOLI KARVVSMPSTDAFDKQDAAYRESVLPKAVTARVAVEAGIADYWYKYVGLN 626

sp|P33570|TKT2_ECOLI NVRVVSLPSTDIFDAQDEEYRESVLPSNVAARVAVEAGIADYWYKYVGLK 625

sp|P45694|TKT_BACSU DVSVVSMPSMDRFEKQSDEYKNEVLPADVKKRLAIEMGSSFGWGKYTGLE 628

tr|A9NEC3|A9NEC3_ACHLI DIRVVSMPSHHAFLLQNEDYRSKILPANVKT-LAVEMGSSYSWYRFTPH- 616

tr|G9XYY6|G9XYY6_SPIME KVKVVSMPCWELFEQQDAEYKQRLLEPLALI-ASIELGTTFGWERYTSNS 614

tr|G9Y039|G9Y039_SPIME NIRVVSMPCVELFEQQPRAYQEKIVPKNITKVIAIEFSNDYVWYKFVGKT 623

tr|Q7NC51|Q7NC51_MYCGA DIDVISVASFKKLKEKLADLEVLKEFKKYKN-IVIESGVSNIWFQFVSL- 610

tr|Q7NAR4|Q7NAR4_MYCGA TARIVSVISLTDFDQINAVSKSVLLKNLPSYFISRADKVAWGYVLSQNQP 593

::*: . : . :

sp|P27302|TKT1_ECOLI GAIVGMTTFGESAPAELLFEEFGFTVDNVVAKAKELL------------- 663

sp|P33570|TKT2_ECOLI GAIVGMTGYGESAPADKLFPFFGFTAENIVAKAHKVLGVKGA-------- 667

sp|P45694|TKT_BACSU GDVLGIDRFGASAPGETIINEYGFSVPNVVNRVKALINK----------- 667

tr|A9NEC3|A9NEC3_ACHLI --VYGIDTFGLSANADVVLKHFGFNKESIAEAFINIK------------- 651

tr|G9XYY6|G9XYY6_SPIME GLNFGTDTFGQSGPFQDVLEYFSFNVEKIVNVINKKMQVL---------- 654

tr|G9Y039|G9Y039_SPIME GLVLGVDDYGLSGSADAVIKYKQLDQSSIINRITAYLEE----------- 662

tr|Q7NC51|Q7NC51_MYCGA --VIGVNDFGLSGNPNEVATYFSMDLESLVKSLASILHKNYTQQKIEELI 658

tr|Q7NAR4|Q7NAR4_MYCGA EVDHKSTENTTYIKKPRRAQKSNNKIKTLDNKTKVDESNKQLNATKEDKQ 643

.:

sp|P27302|TKT1_ECOLI ----

sp|P33570|TKT2_ECOLI ----

sp|P45694|TKT_BACSU ----

tr|A9NEC3|A9NEC3_ACHLI ----

tr|G9XYY6|G9XYY6_SPIME ----

tr|G9Y039|G9Y039_SPIME ----

tr|Q7NC51|Q7NC51_MYCGA KNAN 662

tr|Q7NAR4|Q7NAR4_MYCGA ----

- 1. **Glyceraldehyde-3-phosphate dehydrogenase (GAPDH)**

**Reaction:** D-glyceraldehyde 3-phosphate + phosphate + NAD+ = 3-phospho-D-glyceroyl phosphate + NADH.

There are no significant amino acid substitutions in active site.

tr|Q7NBG8|Q7NBG8_MYCGA MSKIKIGINGFGRIGRLVCRELLNHE-DVEVVAVNDLTDAKTLAYLLKYD 49

tr|Q7NAS3|Q7NAS3_MYCGA --------------------------------------------------

tr|G9XZ04|G9XZ04_SPIME --MTKIAINGFGRIGRLAFRRLFDEK-NVEIVAINDLTEAKTLATLLELD 47

tr|A9NHE5|A9NHE5_ACHLI -MAIKVAINGFGRIGRLAF-RLMVDNPAFDVVAINDLTDTETLAYLLKYD 48

sp|P33898|G3P3_ECOLI --MSKVGINGFGRIGRLVLGRLLEVKSNIDVVAINDLTSPKILAYLLKHD 48

sp|P09124|G3P1_BACSU -MAVKVGINGFGRIGRNVFRAALNNP-EVEVVAVNDLTDANMLAHLLQYD 48

sp|O34425|G3P2_BACSU -MKVKVAINGFGRIGRMVFRKAMLDD-QIQVVAINASYSAETLAHLIKYD 48

sp|P0A9B2|G3P1_ECOLI -MTIKVGINGFGRIGRIVFRAAQKRS-DIEIVAINDLLDADYMAYMLKYD 48

tr|Q7NBG8|Q7NBG8_MYCGA TAHGKLSHN-VSSTDSEIVVGKQKIKVYSEKDPTQIPWKKHKVDLVVEST 98

tr|Q7NAS3|Q7NAS3_MYCGA --------------------------------------------------

tr|G9XZ04|G9XZ04_SPIME SAQGGWKRGKISSEEGVIIVDGKKINVYAKKDPTELPWGKLGIDVVVEST 97

tr|A9NHE5|A9NHE5_ACHLI TAQGPFKGHEVSTNGDKLVVDGKSITVYAQKDPSELPWGTLGVDVVLEST 98

sp|P33898|G3P3_ECOLI SNYGPFP-WSVDFTEDSLIVDGKSIAVYAEKEAKNIPWKAKGAEIIVECT 97

sp|P09124|G3P1_BACSU SVHGKLD-AEVSVDGNNLVVNGKTIEVSAERDPAKLSWGKQGVEIVVEST 97

sp|O34425|G3P2_BACSU TIHGRYD-KEVVAGEDSLIVNGKKVLLLNSRDPKQLPWREYDIDIVVEAT 97

sp|P0A9B2|G3P1_ECOLI STHGRFD-GTVEVKDGHLIVNGKKIRVTAERDPANLKWDEVGVDVVAEAT 97

tr|Q7NBG8|Q7NBG8_MYCGA GRFLTQEAASAHLKGGAKKVVLSAPAKEKNVKTVVYNVNHTEIKPDDTVI 148

tr|Q7NAS3|Q7NAS3_MYCGA ----------------------------------MY------YKP----- 5

tr|G9XZ04|G9XZ04_SPIME GFFADRAGASKHLTAGAKKVLISAPAKGTDVKTIVYNVNHKEIKKEDTII 147

tr|A9NHE5|A9NHE5_ACHLI GLFTSAEKAGLHIKAGAKKVVVSAPATGEGVKTVVYNVNDDILDGTETIV 148

sp|P33898|G3P3_ECOLI GFYTSAEKSQAHLDAGAKKVLISAPAG--EMKTIVYNVNDDTLDGNDTIV 145

sp|P09124|G3P1_BACSU GFFTKRADAAKHLEAGAKKVIISAPANEEDITIVMGVNEDKYDAANHDVI 147

sp|O34425|G3P2_BACSU GKFNAKDKAMGHIEAGAKKVILTAPGKNEDVTIVMGVNEDQFDAERHVII 147

sp|P0A9B2|G3P1_ECOLI GLFLTDETARKHITAGAKKVVMTGPSKDNTPMFVKGANFDKY--AGQDIV 145

tr|Q7NBG8|Q7NBG8_MYCGA SAASCTTNCLAPLVKVLEDKFGIKVGYMTTVHAYTADQRLQDAPH-SDYR 197

tr|Q7NAS3|Q7NAS3_MYCGA ---------LAPVVKVLVEEFGLKSGYMTTVNSYTADQKLQDALH-KDLR 45

tr|G9XZ04|G9XZ04_SPIME SGASCTTNCLAPMAKVLDEKFGIEKGYMTTVHAVTNDQRLLDLAH-DDLR 196

tr|A9NHE5|A9NHE5_ACHLI SAASCTTNALAPLAKVLDDNFGIVKGFMTTVHAYTNDQSLMDQPHKKGFM 198

sp|P33898|G3P3_ECOLI SVASCTTNCLAPMAKALHDSFGIEVGTMTTIHAYTGTQSLVDGPRGKDLR 195

sp|P09124|G3P1_BACSU SNASCTTNCLAPFAKVLNDKFGIKRGMMTTVHSYTNDQQILDLP-HKDYR 196

sp|O34425|G3P2_BACSU SNASCTTNCLAPVVKVLDEEFGIESGLMTTVHAYTNDQKNIDNP-HKDLR 196

sp|P0A9B2|G3P1_ECOLI SNASCTTNCLAPLAKVINDNFGIIEGLMTTVHATTATQKTVDGPSHKDWR 195

***..*.: :.**: * ***::: * * * ..

tr|Q7NBG8|Q7NBG8_MYCGA RGR--AAAFNMVPTSTGAAKAIGLVVPKATGKLNGIAVRVPTITGSLVDL 245

tr|Q7NAS3|Q7NAS3_MYCGA RAR--AAEN-IVSTSTGAAKAIGLVVPQASNVLDGIAIIVPTLTGSLIDL 92

tr|G9XZ04|G9XZ04_SPIME RAR--AAFSNIVPTKTGAAAAVALVLPQLEGRFDGMALRVPTITGSIVDL 244

tr|A9NHE5|A9NHE5_ACHLI SRRGRAAAASIIPSSTGAAAAIGLVLPQLKGKLDGTALRVPTITGSIVDL 248

sp|P33898|G3P3_ECOLI ASR--AAAENIIPHTTGAAKAIGLVIPELSGKLKGHAQRVPVKTGSVTEL 243

sp|P09124|G3P1_BACSU RAR--AAAENIIPTSTGAAKAVSLVLPELKGKLNGGAMRVPTPNVSLVDL 244

sp|O34425|G3P2_BACSU RAR--ACGESIIPTTTGAAKALSLVLPHLKGKLHGLALRVPVPNVSLVDL 244

sp|P0A9B2|G3P1_ECOLI GGR--GASQNIIPSSTGAAKAVGKVLPELNGKLTGMAFRVPTPNVSVVDL 243

* .. ::. .**** *:. *:*. . : * * **. . *: :*

tr|Q7NBG8|Q7NBG8_MYCGA TVKLEKDTTVEKINAAMKAAAS----ESFLYSEDEIVSSDILNETHGSIF 291

tr|Q7NAS3|Q7NAS3_MYCGA SVRLEKSPSVEELNASFKKAAN----ESFKFETDEIVSSDIVNSHYGSVF 138

tr|G9XZ04|G9XZ04_SPIME AVELKKTTTVEEINNAMKAAAS----ETFGYNTQPIVSSDIIGETHGSIF 290

tr|A9NHE5|A9NHE5_ACHLI TVELGKEVTLAEIDAAFKAAAN----ESLQYQPDPIVSADVIGSHFGSVY 294

sp|P33898|G3P3_ECOLI VSILGKKVTAEEVNNALKQATTN--NESFGYTDEEIVSSDIIGSHFGSVF 291

sp|P09124|G3P1_BACSU VAELNQEVTAEEVNAALKEAAEGDLKGILGYSEEPLVSGDYNGNKNSSTI 294

sp|O34425|G3P2_BACSU VVDLKTDVTAEEVNEAFKRAAKTSMYGVLDYSDEPLVSTDYNTNPHSAVI 294

sp|P0A9B2|G3P1_ECOLI TVRLEKAATYEQIKAAVKAAAEGEMKGVLGYTEDDVVSTDFNGEVCTSVF 293

* : ::. :.* *: : : : :** * . :

tr|Q7NBG8|Q7NBG8_MYCGA DSKLTSVLETNGEKLYKLYAWYDNESSYVSQLVRVAKYFAKQQPKKASAK 341

tr|Q7NAS3|Q7NAS3_MYCGA DSKLTNFVESKDGRLYKLFAWYDNEMSYVSQLVRVLEYFVSL-------- 180

tr|G9XZ04|G9XZ04_SPIME DATLTKIIERDGKQLVKVYAWYDNEMSYVSQMVRTLLYFATV-------- 332

tr|A9NHE5|A9NHE5_ACHLI DANTTQILKT-DAKFVKVMGWYDNEMSYTAQLIRVMAKIANLTK------ 337

sp|P33898|G3P3_ECOLI DATQTEITAVGDLQLVKTVAWYDNEYGFVTQLIRTLEKFAKL-------- 333

sp|P09124|G3P1_BACSU DALSTMVMEG---SMVKVISWYDNESGYSNRVVDLAAYIAKKGL------ 335

sp|O34425|G3P2_BACSU DGLTTMVMED---RKVKVLAWYDNEWGYSCRVVDLIRHVAARMKHPSAV- 340

sp|P0A9B2|G3P1_ECOLI DAKAGIALND---NFVKLVSWYDNETGYSNKVLDLIAHISK--------- 331

*. * .***** .: ::: .

tr|Q7NBG8|Q7NBG8_MYCGA K 342

tr|Q7NAS3|Q7NAS3_MYCGA -

tr|G9XZ04|G9XZ04_SPIME -

tr|A9NHE5|A9NHE5_ACHLI -

sp|P33898|G3P3_ECOLI -

sp|P09124|G3P1_BACSU -

sp|O34425|G3P2_BACSU -

sp|P0A9B2|G3P1_ECOLI -

- 1. **Glyceraldehyde-3-phosphate dehydrogenase (GAPN)**

**Reaction:** D-glyceraldehyde 3-phosphate + NADP+ + H2O = 3-phospho-D-glycerate + NADPH.

Here we used the crystal structure of the GapN protein from *Streptococcus mutans* as the standard.

For *A. laidlawii* it was annotated two GAPN coding genes and the both corresponding proteins (**A9NHU4_ACHL; A9NHU7_ACHLI**) have amino acid substitutions: R103Y; S151Y; T180E; D215K; R437H.

tr|Q7NBX5|Q7NBX5_MYCGA MSNSECIKKKVCNASSYDFKDKYHHLKGLINNQEVGSSKLIEIKSPIDHE 50

tr|G9XZV7|G9XZV7_SPIME ---------------------MNLELNALINGELINNGDWLEIISPINNK 29

sp|Q59931|GAPN_STRMU ---------------------MTKQYKNYVNGEWKLSENEIKIYEPASGA 29

tr|A9NHU4|A9NHU4_ACHLI --------------------------------------------------

tr|A9NHU7|A9NHU7_ACHLI --------------------------------------------------

tr|Q7NBX5|Q7NBX5_MYCGA LSGSFYGMTAEEIDDAYEKADIAFKSWSKAGYEYRKAKILKFAQLLDAHK 100

tr|G9XZV7|G9XZV7_SPIME PYGRVPALKEKEINEAFKGARHSQKAWAKLTLFERISYLQKWAKLLESHK 79

sp|Q59931|GAPN_STRMU ELGSVPAMSTEEVDYVYASAKKAQPAWRSLSYIERAAYLHKVADILMRDK 79

tr|A9NHU4|A9NHU4_ACHLI ------------METIVQSQKTLFLSGKTLSYEFRIRQLQKLKQLIINYQ 38

tr|A9NHU7|A9NHU7_ACHLI ------------MDGMMIG-----LIQTTVLYGATHRNHDK--------- 24

:: . *

tr|Q7NBX5|Q7NBX5_MYCGA EEFANLLTDNIAKA-YNESLAEVTRSIQYIHDTISVYEEMITHPLVMDEK 149

tr|G9XZV7|G9XZV7_SPIME TELAKIMAHEVGKS-LKDGQIEVERSIEYIDYTIEEAKRVFPETLTGDG- 127

sp|Q59931|GAPN_STRMU EKIGAVLSKEVAKG-YKSAVSEVVRTAEIINYAAEEGLRMEGEVLEGGSF 128

tr|A9NHU4|A9NHU4_ACHLI DEILEALFKDLHKSRFEAYSTEVGYVLKSLTHTIKGLKKWMKPKKVKTP- 87

tr|A9NHU7|A9NHU7_ACHLI -----SIYEDLHKSAFESYSTEIGYVLKSITKTIKSLKKWMKPKRVKTP- 68

: .:: *. : *: : : : . .

tr|Q7NBX5|Q7NBX5_MYCGA IHRVKEKVGKFVREPLGVALNISPWNYPLNTPLSKMIPTLIAGNTVVYKV 199

tr|G9XZV7|G9XZV7_SPIME -WNVKNKIGIFSRVPKGVVLAISPFNYPVNLSIAKIAPSLVVGNTVVFKP 176

sp|Q59931|GAPN_STRMU EAASKKKIAVVRREPVGLVLAISPFNYPVNLAGSKIAPALIAGNVIAFKP 178

tr|A9NHU4|A9NHU4_ACHLI -YYLSATSSYITYDALGTILIIGPYNYPFQLIIEPLIGAIAAGNTVMIKP 136

tr|A9NHU7|A9NHU7_ACHLI -IYLRPTTSYIAYEPKGVILIIGPYNYPFQLILEPLIGAIAAGNTASIKP 117

. . . . * * *.*:***.: : :: .**. *

tr|Q7NBX5|Q7NBX5_MYCGA ATQAAIIGIRLAHLFKQAGFDPGVVQCVVGLGREIGDKLNTNKHIKSISF 249

tr|G9XZV7|G9XZV7_SPIME ATNGSLTGLYMAKLAFEVGFPKGVFNVVTGRGRDIGDLLVLNPEINVISF 226

sp|Q59931|GAPN_STRMU PTQGSISGLLLAEAFAEAGLPAGVFNTITGRGSEIGDYIVEHQAVNFINF 228

tr|A9NHU4|A9NHU4_ACHLI SEFATYTEKILVKLVNNH-FDKDYLYVIEGDYTVTSKLLDS--KFDHIFF 183

tr|A9NHU7|A9NHU7_ACHLI SEYASHTEQLLQTLINDH-FEPSYLHVITGDHKITENLLEN--KFDHIFF 164

. .: : : : . . : * . : .. * *

tr|Q7NBX5|Q7NBX5_MYCGA TGSTPVGLSLLKQS--AVGNISLELGGKDAALVLSDYDKQKTIKEIVKGA 297

tr|G9XZV7|G9XZV7_SPIME TGSVAVGNHIRKLG--HGKDLVLELGGKDPALVLKDADLAKAVKEIVAGA 274

sp|Q59931|GAPN_STRMU TGSTGIGERIGKMA--GMRPIMLELGGKDSAIVLEDADLELTAKNIIAGA 276

tr|A9NHU4|A9NHU4_ACHLI TGSSRVGQIVYEKASKHLTPVTLELGGKSPTIVDETANLKIAAERILFGK 233

tr|A9NHU7|A9NHU7_ACHLI TGSTRVGKLIYEQASKHLTPVTLELGGKSPTIVDQSANLKVAAKRIIFGK 214

*** :* : : . : ******..::* . : : :.*: *

tr|Q7NBX5|Q7NBX5_MYCGA YSYSGQRCTAIKRVFVKNEDADEFVQLLKKEVEKLHLGNPFNNPSLVPVI 347

tr|G9XZV7|G9XZV7_SPIME YAYSGQRCTAIKRVLVDETIAAELVQLLKIEVGKLEVGSPLDNKAIIPVI 324

sp|Q59931|GAPN_STRMU FGYSGQRCTAVKRVLVMESVADELVEKIREKVLALTIGNPEDDADITPLI 326

tr|A9NHU4|A9NHU4_ACHLI FLNAGQTCIAPDYIYVHDTIHDEFINILNQVINTRYS----DMNYFGRII 279

tr|A9NHU7|A9NHU7_ACHLI FINAGQTCIAPDYIYVHQTIAESFIKILSDTMHTMYN----DLHQFGRII 260

: :** * * . : * : .::: : : : : :*

tr|Q7NBX5|Q7NBX5_MYCGA DQHAANFIKELFDDAVTKKAIVLTGGDFEKNCVEAILLDHVTTDMRVAWE 397

tr|G9XZV7|G9XZV7_SPIME DLKAADFVQGLIDDALAKKATLVLGNQRKDNLLSATLIDHVTPEMRLAWE 374

sp|Q59931|GAPN_STRMU DTKSADYVEGLINDANDKGAAALTEIKREGNLICPILFDKVTTDMRLAWE 376

tr|A9NHU4|A9NHU4_ACHLI NERHYKRLIGLIDET---KMIQKPTVEAETKLISPTVLIDVTWDDKVMQE 326

tr|A9NHU7|A9NHU7_ACHLI NDKHFDRLMNLIDAD---KVLQKPVIDAKMCLISPTIMKGVTWTDKVMQE 307

: : . : *:: . : : . :: ** :: *

tr|Q7NBX5|Q7NBX5_MYCGA EPFGPILPIIRVKNEAEMVKLHNQSEYGLQASIFTSDQTKFDELANQLEA 447

tr|G9XZV7|G9XZV7_SPIME EPFGPVLPIIRCKNVEEMITLANKSNFKLQACIFTKDINAAFNIANELET 424

sp|Q59931|GAPN_STRMU EPFGPVLPIIRVTSVEEAIEISNKSEYGLQASIFTNDFPRAFGIAEQLEV 426

tr|A9NHU4|A9NHU4_ACHLI EIFGPILPVLKYANLKDLVSLLKTKDKPLALYLFSNDKKNQTYVFENLTF 376

tr|A9NHU7|A9NHU7_ACHLI EIFGPLLPVLIYDDIDTLIKLLKTKEKPLALYVFTNNKDIQHKVFSQLSF 357

* ***:**:: . : : : .: * :*:.: : .:*

tr|Q7NBX5|Q7NBX5_MYCGA GTINWNRSSSRGPD-FFPFMGVKDSGVGVQGIRDTILSVTRYKGFVYNR- 495

tr|G9XZV7|G9XZV7_SPIME GTVNINGRTQRGPD-SFPFLGIHDSGQGVQGIRETINSVTRFKGLVINY- 472

sp|Q59931|GAPN_STRMU GTVHINNKTQRGTD-NFPFLGAKKSGAGIQGVKYSIEAMTTVKSVVFDIK 475

tr|A9NHU4|A9NHU4_ACHLI GGGAINDTIMHVSNPYLPFGGIGMSGIGAYHGFYSFKLFSHTKGYVKKAT 426

tr|A9NHU7|A9NHU7_ACHLI GGGAMNDTIMHVSNPCLPFGGVGQSGMGAYHYFASFEIFSHKKSYIKRCT 407

* * : .: :** * ** * :: .: *. :

tr|Q7NBX5|Q7NBX5_MYCGA ------------------------

tr|G9XZV7|G9XZV7_SPIME ------------------------

sp|Q59931|GAPN_STRMU ------------------------

tr|A9NHU4|A9NHU4_ACHLI WFDLPIIYPPYTKFKEKLIRKIFK 450

tr|A9NHU7|A9NHU7_ACHLI WLYLPLVYPPYTKSKENIIKKILK 431

- 1. **Enolase**

There are no significant amino acid substitutions in the active site.

sp|A9NF93|ENO_ACHLI -----------MPFITSVYAREVLDSRGNPTVEVEIVTDSGAFGRTLVPS 39

sp|P37869|ENO_BACSU -----------MPYIVDVYAREVLDSRGNPTVEVEVYTETGAFGRALVPS 39

sp|P0A6P9|ENO_ECOLI -----------MSKIVKIIGREIIDSRGNPTVEAEVHLEGGFVGMAAAPS 39

tr|G9XX91|G9XX91_SPIME -----------MSRIEKINAREVIDSRGNPTVQVEVWTEFGGYGSAMVPS 39

sp|Q7NAY0|ENO_MYCGA MAKTNSTSKNNKLEIKSVFAYQAFDSRGFPTVACEVVLNDGSKGLSMVSS 50

* .: . : :**** *** *: : * * : ..*

sp|A9NF93|ENO_ACHLI GASTGEHEAVELRDGDKSRYLGKGVLKAVENVNDIIGPAILGDSVLDQVA 89

sp|P37869|ENO_BACSU GASTGEYEAVELRDGDKDRYLGKGVLTAVNNVNEIIAPELLGFDVTEQNA 89

sp|P0A6P9|ENO_ECOLI GASTGSREALELRDGDKSRFLGKGVTKAVAAVNGPIAQALIGKDAKDQAG 89

tr|G9XX91|G9XX91_SPIME GASTGSREALELRDGDKGRYQGKGVLKAVGNVNTKIAEQLVGMEVTNQVE 89

sp|Q7NAY0|ENO_MYCGA GASTGEKEALELRDGG-TKYHGKGVTKAVNNINKKIGPKILGVDATLQTQ 99

*****. **:*****. :: **** .** :* *. ::* .. *

sp|A9NF93|ENO_ACHLI LDRKLIALDGTKNKGKLGANAILGVSIAAAKAAADLLNLELYQYLGGFN- 138

sp|P37869|ENO_BACSU IDQLLIELDGTENKGKLGANAILGVSMACARAAADFLQIPLYQYLGGFN- 138

sp|P0A6P9|ENO_ECOLI IDKIMIDLDGTENKSKFGANAILAVSLANAKAAAAAKGMPLYEHIAELNG 139

tr|G9XX91|G9XX91_SPIME IDHAMIALDGTDFKKNLGANAMLGVSMAVAKAAASELEMPLYKYLGGVN- 138

sp|Q7NAY0|ENO_MYCGA IDEFMIELDGTKTKAKLGANAILAVSMAVCRAAAKSLNLPLYQYIAKKVA 149

:*. :* ****. * ::****:*.**:* .:*** : **:::.

sp|A9NF93|ENO_ACHLI -----AKQLPVPMMNIINGGAHSDAPIDFQEFMIFPVGAPSFKEAIRWGA 183

sp|P37869|ENO_BACSU -----SKTLPVPMMNIVNGGEHADNNVDIQEFMIMPVGAPNFREALRMGA 183

sp|P0A6P9|ENO_ECOLI TPG--KYSMPVPMMNIINGGEHADNNVDIQEFMIQPVGAKTVKEAIRMGS 187

tr|G9XX91|G9XX91_SPIME -----GKKLPVPMLNIINGGEHADSAIDFQEFMIMPVGAETFKEALRWSS 183

sp|Q7NAY0|ENO_MYCGA KVKGADFILPVPMLNVINGGAHADNTIDFQEFMIMPVGAKTMAKALQMAS 199

:****:*::*** *:* :*:***** **** .. :*:: .:

sp|A9NF93|ENO_ACHLI EIFHALKAILKKKGLSTAVGDEGGFAPNL---------------ASNEDT 218

sp|P37869|ENO_BACSU QIFHSLKSVLSAKGLNTAVGDEGGFAPNL---------------GSNEEA 218

sp|P0A6P9|ENO_ECOLI EVFHHLAKVLKAKGMNTAVGDEGGYAPNL---------------GSNAEA 222

tr|G9XX91|G9XX91_SPIME EIFHTLKKILHDKGDITAVGDEGGFAPHFNWAYKDQTLTSFQAKTPAEVA 233

sp|Q7NAY0|ENO_MYCGA EVFHSLQKLLKAKKFNTNKGDEGGFAPNL---------------KSAEEA 234

::** * :* * * *****:**:: . :

sp|A9NF93|ENO_ACHLI IDNILEAIKNAGYKAGEE-VFIGFDVAASEFFDKAKGKYVFKK------- 260

sp|P37869|ENO_BACSU LQTIVEAIEKAGFKPGEE-VKLAMDAASSEFYNKEDGKYHLS-------- 259

sp|P0A6P9|ENO_ECOLI LAVIAEAVKAAGYELGKD-ITLAMDCAASEFYK--DGKYVLAG------- 262

tr|G9XX91|G9XX91_SPIME LDLIVEAIEAAGYKPGKDGVMIAMDCASSELYFD-DKKYHFKKIEKV--- 279

sp|Q7NAY0|ENO_MYCGA LDLMSQAVVDAGYALGKD-VAFALDCAASEFYSKEKQAYVFKKAVKAGIL 283

: : :*: **: *:: : :.:* *:**:: . * :

sp|A9NF93|ENO_ACHLI -STKEEFTSAELVEYYAGLVQKYPIISIEDGMDENDWDGWKLLTDKLGSK 309

sp|P37869|ENO_BACSU -GEGVVKTSAEMVDWYEELVSKYPIISIEDGLDENDWEGHKLLTERLGKK 308

sp|P0A6P9|ENO_ECOLI -EGNKAFTSEEFTHFLEELTKQYPIVSIEDGLDESDWDGFAYQTKVLGDK 311

tr|G9XX91|G9XX91_SPIME TGKEYAMTTEELIKYLDKLVDKYPIISIEDGLAEGDWAGFELQVKTMGHK 329

sp|Q7NAY0|ENO_MYCGA SEEKGTKTTEQLISYLEDLTKKYPIVSIEDGLDENDWKGMESLTKKIGKK 333

*: :: : *..:***:*****: *.** * .. :* *

sp|A9NF93|ENO_ACHLI IQLVGDDLFVTNTEYLARGIKTNTANSILVKVNQIGTLTETFDAIEMAKR 359

sp|P37869|ENO_BACSU VQLVGDDLFVTNTKKLSEGIKNGVGNSILIKVNQIGTLTETFDAIEMAKR 358

sp|P0A6P9|ENO_ECOLI IQLVGDDLFVTNTKILKEGIEKGIANSILIKFNQIGSLTETLAAIKMAKD 361

tr|G9XX91|G9XX91_SPIME IQIVGDDLFVTNPKITAEGIARNAANSVLIKLNQIGTVTETIDTIQLAQK 379

sp|Q7NAY0|ENO_MYCGA VQIVGDDTYCTNPELTSKGVSLSATNSVLIKLNQIGTLTETIQTINIAKK 383

:*:**** : **.: .*: . **:*:*.****::***: :*::*:

sp|A9NF93|ENO_ACHLI AGYTAVISHRSGETEDTTIADIAVAANTGQIKTGSASRTDRVAKYNQLMR 409

sp|P37869|ENO_BACSU AGYTAVISHRSGETEDSTIADIAVATNAGQIKTGAPSRTDRVAKYNQLLR 408

sp|P0A6P9|ENO_ECOLI AGYTAVISHRSGETEDATIADLAVGTAAGQIKTGSMSRSDRVAKYNQLIR 411

tr|G9XX91|G9XX91_SPIME AGWTAVVSHRSGETEDSTIADLAVALNTGQIKTGSMSRSDRIAKYNRLLA 429

sp|Q7NAY0|ENO_MYCGA ANWTAVVSHRSGETEDAFIADLAVALSTGQIKTGSMSRSERIAKYNRLLA 433

*.:***:*********: ***:**. :******: **::*:****:*:

sp|A9NF93|ENO_ACHLI IEDQLGEDAVYLGKDSFYNLKK-------------------- 431

sp|P37869|ENO_BACSU IEDQLAETAQYHGINSFYNLNK-------------------- 430

sp|P0A6P9|ENO_ECOLI IEEALGEKAPYNGRKEIKGQA--------------------- 432

tr|G9XX91|G9XX91_SPIME IEDELGKVAEYDGIKTFYNLKKHVADLKK------------- 458

sp|Q7NAY0|ENO_MYCGA IEMQLGNKAKYLGSKTFYNLSTPAATPKKSPAKKTTKAKSKK 475

- 1. **Tdk. Thymidine kinase**

We found (relative to *B. subtilis*) next substitutions in substrate binding sites: V89A, C148T, C183S, and H186A in A9NEC0_ACHLI protein for *A. lailawii*; and 186 H->C in G9XYA3_SPIME protein for *S. melliferum*.

tr|A9NEC0|A9NEC0_ACHLI MYHTYRN----GFIEVVCGPMFAGKTEELIRRAKRLKYAKQNYLVFKPVI 46

tr|G9XYA3|G9XYA3_SPIME MYFLNSKAQI-GWIEVITGCMFAGKTEEFIRRLVRLSYAKFEIQVFKPTI 49

sp|Q03221|KITH_BACSU MYIMKQS----GWLELICGSMFSGKSEELIRRVKRATYAKQEVRVFKPVI 46

sp|Q8RLE0|KITH_MYCGA MAKKNAMTTTNGWIEAICGPMFAGKTDELIRKIKRYEYADVKSLVFSPAT 50

sp|P23331|KITH_ECOLI ----------MAQLYFYYSAMNAGKSTALLQSSYNYQERGMRTVVYTAEI 40

. : . * :**: ::: . . *:..

tr|A9NEC0|A9NEC0_ACHLI DNRYSSKAEIVSHSLLTENAILIEKSKDILEH-----MNDSIEAVIIDEA 91

tr|G9XYA3|G9XYA3_SPIME DNRYS-ENQVVSHSKKAVEAISVKDSDELLAN-----LKTTTNVVGIDEV 93

sp|Q03221|KITH_BACSU DNRYS-EAAVVSHNGTSMTSYAISSAADIWDH-----ISESTDVVAVDEV 90

sp|Q8RLE0|KITH_MYCGA DTRSA-QEIINSRDGRRIGSIKIKKAFEIYDYV----LLHKPQLVGIDEV 95

sp|P23331|KITH_ECOLI DDRFG-AGKVSSRIGLSSPAKLFNQNSSLFDEIRAEHEQQAIHCVLVDEC 89

* * . : *: : ... .: . * :**

tr|A9NEC0|A9NEC0_ACHLI QFFD-QDIVEIADRLADSGVRVIIGGLDRDFKGEPFGPMPQLLAIAEFVV 140

tr|G9XYA3|G9XYA3_SPIME QFFD-NNIVKIADSLADKGIIVIVNGLDKDFRGEAFTNIEQLMTRAEEVK 142

sp|Q03221|KITH_BACSU QFFD-QEIVEVLSSLADKGYRVIAAGLDMDFRGEPFGVVPNIMAIAESVT 139

sp|Q8RLE0|KITH_MYCGA QFFD-DSIVEVIQTLADNQINVIVAGLDRDFRGEPFGPIPKILGIAESVI 144

sp|P23331|KITH_ECOLI QFLTRQQVYELSEVVDQLDIPVLCYGLRTDFRGELFIGSQYLLAWSDKLV 139

**: :.: :: . : : *: ** **:** * :: :: :

tr|A9NEC0|A9NEC0_ACHLI KLTAICPKTGTPATRTQRIINGKPARMDDPLIVVGASDSYEPRSRHAHEV 190

tr|G9XYA3|G9XYA3_SPIME KLHAICVKCGNLANRTQRLINGKPANYYDPIVLIGEKDKYEARCRHCHEV 192

sp|Q03221|KITH_BACSU KLQAVCSVCGSPASRTQRLIDGKPASYDDPVILVGAAESYEARCRHHHEV 189

sp|Q8RLE0|KITH_MYCGA RLTAICSECGAEASRSQRLIDNQPADYNCETILIGDTESYAPRCRHHHKV 194

sp|P23331|KITH_ECOLI ELKTIC-FCGRKASMVLRLDQAGRPYNEGEQVVIGGNERYVSVCRKHYKE 188

.* ::* * *. *: : . :::* : * . .*: ::

tr|A9NEC0|A9NEC0_ACHLI PGKYDKK----------------------- 197

tr|G9XYA3|G9XYA3_SPIME T--Y-------------------------- 194

sp|Q03221|KITH_BACSU PGKSKK------------------------ 195

sp|Q8RLE0|KITH_MYCGA PNRPINDQTKNFKRALKNNFDKIVEQSSKD 224

sp|P23331|KITH_ECOLI ALQVDSLTAIQERHRHD------------- 205

.

- 1. **Udk. Uridine kinase**

Rections:

ATP + cytidine = ADP + CMP

ATP + uridine = ADP + UMP

Here we used the crystal structure of the Udk protein from *Thermus thermophilus* as a standard. We found (relative to *T. thermophilus*) the next substitutions in substrate binding sites: A16G in Q7NB32_MYCGA protein for *M. gallisepticum* and Y159F in G9Y068_SPIME protein for *S. melliferum.*

sp|O32033|URK_BACSU ----MGKNPVVIGIAGGSGSGKTSVTRSIY----EQFKGHSILMIQQDLY 42

sp|P0A8F4|URK_ECOLI -MTDQSHQCVIIGIAGASASGKSLIASTLYRELREQVGDEHIGVIPEDCY 49

sp|Q5SKR5|URK_THET8 ---MSAPKPFVIGIAGGTASGKTTLAQALA-----RTLGERVALLPMDHY 42

tr|A9NHB4|A9NHB4_ACHLI -----MKKPFFMIVAGGSASGKSTVVKSIL----EKAGIQHVLVINQDDY 41

tr|G9Y068|G9Y068_SPIME MQPKPKNKVQLVTITGGTASGKTTVANKIA----EILQGKKIVYLKMDHY 46

tr|Q7NB32|Q7NB32_MYCGA ---MKNVQPMIIAIAGGSGSGKTTITQSIIAK-IKSDTNLKVATVCLDNY 46

: .: ::*.:.***: :. : : : * *

sp|O32033|URK_BACSU YKDQSHLPFEERLNTNYDHPLAFDNDYLIEHIQDLLNYRPIEKPIYDYKL 92

sp|P0A8F4|URK_ECOLI YKDQSHLSMEERVKTNYDHPSAMDHSLLLEHLQALKRGSAIDLPVYSYVE 99

sp|Q5SKR5|URK_THET8 YKDLGHLPLEERLRVNYDHPDAFDLALYLEHAQALLRGLPVEMPVYDFRA 92

tr|A9NHB4|A9NHB4_ACHLI YLDQNELPMHERVLMNYDHPKSIDIELLKKDILMLLEGKTIHKPTYDYSN 91

tr|G9Y068|G9Y068_SPIME YKKLDDLTLAERKKINFDHPNALDLELLVNHLQLLKNHQNIQMPNYDFTI 96

tr|Q7NB32|Q7NB32_MYCGA YKPFSDLNLEARKKLNYDDPNSFDFDQVYYDLLALSNNQTIKMPIYDYKN 96

* ..* : * *:*.* ::* . * . :. * *.:

sp|O32033|URK_BACSU HTRSEETVHVEPKDVIILEGILVLEDKRLRDLMDIKLYVDTDADLRIIRR 142

sp|P0A8F4|URK_ECOLI HTRMKETVTVEPKKVIILEGILLLTDARLRDELNFSIFVDTPLDICLMRR 149

sp|Q5SKR5|URK_THET8 YTRSPRRTPVRPAPVVILEGILVLYPKELRDLMDLKVFVDADADERFIRR 142

tr|A9NHB4|A9NHB4_ACHLI YTRSEVIEEVESKPIIILEGILSLVDDSIRDLADLKLYVELDDDIRFIRR 141

tr|G9Y068|G9Y068_SPIME SNRSIQTTEIKSGDVIILDGILGLALEEIRKLSDIKIFIKTEDDIRFIRR 146

tr|Q7NB32|Q7NB32_MYCGA YTRSDQFTEIDPSDVILYEGILSLYDSRILDLSKFKLFIDTPSDERLARR 146

.* : . ::: :*** * : . .:.:::. * : **

sp|O32033|URK_BACSU IMRDINERGRSIDSVIEQYVSVVRPMHNQFVEPTKRYADIIIPEGGQNHV 192

sp|P0A8F4|URK_ECOLI IKRDVNERGRSMDSVMAQYQKTVRPMFLQFIEPSKQYADIIVPRGGKNRI 199

sp|Q5SKR5|URK_THET8 LKRDVLERGRSLEGVVAQYLEQVKPMHLHFVEPTKRYADVIVPRGGQNPV 192

tr|A9NHB4|A9NHB4_ACHLI LSRDVKDRGRTMQSVINQYITTVKPMYHKFVKPTKRYADIIIPNDDKHSA 191

tr|G9Y068|G9Y068_SPIME LTRDLSERGRTVESIINQYLTTVKPMHEYFVEPSIKYADIIVPYYEGNEI 196

tr|Q7NB32|Q7NB32_MYCGA IERDCLERARDIKQVLNQWRSQVRVMHRKYVQKQKEGANLILPWYTLNHE 196

: ** :*.* :. :: *: *: *. ::: . *::*:* :

sp|O32033|URK_BACSU AIDLMVTKIQTILEQNAIL 211

sp|P0A8F4|URK_ECOLI AIDILKAKISQFFE----- 213

sp|Q5SKR5|URK_THET8 ALEMLAAKALARLARMGAA 211

tr|A9NHB4|A9NHB4_ACHLI AVDVIVNMLKTVKESI--- 207

tr|G9Y068|G9Y068_SPIME AIDMIATKIKALLLDKK-- 213

tr|Q7NB32|Q7NB32_MYCGA GMSIIQNAIIKIAQGNEF- 214

.:.::

- 1. **Uridylate kinase. PyrH**

**Rection:** ATP + UMP = ADP + UDP

We found (relative to *B. subtilis*) N137S substitution in A9NHC4_ACHLI protein for *A. laidlawii* and N137Q substitution in G9XZS5_SPIME protein for *S. melliferum*.

tr|A9NHC4|A9NHC4_ACHLI -------MYRRIILKISGEALK-GDGQYGIDPKTVKKIAQEIKTVRNAGI 42

sp|O31749|PYRH_BACSU ---MEKPKYKRIVLKLSGEALA-GEQGNGINPTVIQSIAKQVKEIAELEV 46

sp|P0A7E9|PYRH_ECOLI MATNAKPVYKRILLKLSGEALQ-GTEGFGIDASILDRMAQEIKELVELGI 49

tr|G9XZS5|G9XZS5_SPIME ----MALQYKRVLLKLSGEALG-NSDD-LYDAKKIYDIAKQIVKLQKEGL 44

sp|Q7NC20|PYRH_MYCGA ------MQKPNIIIKISGASLQDKNSNDCYSYQRINSLADQLKSLAK-KY 43

.:::*:** :* . : :*.:: : :

tr|A9NHC4|A9NHC4_ACHLI EVAVVVGAGNLWRGKTGEELGMDRAQADYMGMLGTIMNSLALQDALEAIE 92

sp|O31749|PYRH_BACSU EVAVVVGGGNLWRGKTGSDLGMDRATADYMGMLATVMNSLALQDSLETLG 96

sp|P0A7E9|PYRH_ECOLI QVGVVIGGGNLFRGAGLAKAGMNRVVGDHMGMLATVMNGLAMRDALHRAY 99

tr|G9XZS5|G9XZS5_SPIME QIAIVVGGGNIWRGNRADTIKMNPISADYMGMLATVMNALALEAVLKNEG 94

sp|Q7NC20|PYRH_MYCGA NIGLIVGGGNIFRGKLAKDFGVEINKADYIGMLATVINSTLLESKLQSLG 93

::.:::*.**::** :: .*::***.*::*. :. *.

tr|A9NHC4|A9NHC4_ACHLI SPSRVMTA-LQIAAVAEPYIRRRALRHFEKGRVVILAGGTGSPYFSTDTT 141

sp|O31749|PYRH_BACSU IQSRVQTS-IEMRQVAEPYIRRKAIRHLEKKRVVIFAAGTGNPYFSTDTT 145

sp|P0A7E9|PYRH_ECOLI VNARLMSA-IPLNGVCDSYSWAEAISLLRNNRVVILSAGTGNPFFTTDSA 148

tr|G9XZS5|G9XZS5_SPIME SQNVVVTSKIQVPEVASPYLFKKAKAALEKGAIVIMAGGTGQPKFTTDTA 144

sp|Q7NC20|PYRH_MYCGA LKTKVLSA-LEVKGLTNEINPKSLAEVFSDCQIAFFSGGTGNSHFTTDTA 142

: :: : : : . : . :.:::.***.. *:**::

tr|A9NHC4|A9NHC4_ACHLI AALRAAELDSDVILMAKNGVDGVYDKDPNKFSDAVLLREVSHQQVLEKNL 191

sp|O31749|PYRH_BACSU AALRAAEIEADVILMAKNNVDGVYNADPRKDESAVKYESLSYLDVLKDGL 195

sp|P0A7E9|PYRH_ECOLI ACLRGIEIEADVVLKATK-VDGVFTADPAKDPTATMYEQLTYSEVLEKEL 197

tr|G9XZS5|G9XZS5_SPIME ATIRAIEIDADVMLMAKNGVDGIYDKDPRHNADAVRFDNISLNELQKKQL 194

sp|Q7NC20|PYRH_MYCGA TVLRAIQINAQLVLIGKDGVDGVYTDDPKKNKKAKFIEQITYQQALNDQL 192

: :*. :::::::* ... ***:: ** : * .:: : :. *

tr|A9NHC4|A9NHC4_ACHLI QIMDSTAASLCKDNHIEILVFDMNKPGNILKAAKGEDIGTVVRNGANS 239

sp|O31749|PYRH_BACSU EVMDSTASSLCMDNDIPLIVFSIMEEGNIKRAVIGESIGTIVRGK--- 240

sp|P0A7E9|PYRH_ECOLI KVMDLAAFTLARDHKLPIRVFNMNKPGALRRVVMGEKEGTLITE---- 241

tr|G9XZS5|G9XZS5_SPIME KVMDLTASSLAMEDDVKIVVFDINEPDNIYKAAHGNARSTIVTGGKK- 241

sp|Q7NC20|PYRH_MYCGA RVMDLTAFSLAKDHNLKLLIFNIEAEQSIIKTIENKNKHTKITN---- 236

.:** :* :*. :..: : :*.: : :. .: * :

Metabolites accumulation data were juxtaposed with the results of amino acid substitution analysis of enzymes active sites. We propose the effect of such substitutions on the enzymes activity. Active sites of enzymes were determined in accordance with the available data on their crystal structure (e.g., *E. coli* and *B. subtilis*). We indicate mutations that may affect enzyme’s activity.

Glyceraldehyde-3-phosphate is accomulated in *M. gallisepticum* and *S. melliferum.* **Fructose-bisphosphate aldolase catalyzes its generation. A** substitution were detected in the binding site of this enzyme (V50A in Q7NAJ0_MYCGA and G9XY54_SPIME see 5.6), since we can propose that this substitution inrcreases its activity. Usually mutation in the active center of enzyme has an inhibitory effect on the enzyme function and it is true for the most analyzed enzymes (see some examples bellow, all such substitutions are indicated in aligned sequences). In contrast, for **fructose-bisphosphate aldolase** (*M. gallisepticum* and *S. melliferum*), we suppose that amino acid substitution increases its enzymatic activity.

Glyceraldehyde-3-phosphate dehydrogenase (GapN or Gap) is responsible for glyceraldehyde-3-phosphate transformation to phosphoglycerate. Accomulation of glyceraldehyde-3-phosphate in *M. gallisepticum* and *S. melliferum* indicate that Gap (GapN) activity is possibly decreased. There are no mutations in sequences of glyceraldehyde-3-phosphate dehydrogenase in *M. gallisepticum* and *S. melliferum* (both GapN and Gap types) relatively to *Streptococcus mutans* active site sequence. However we detected numerous substitutions in *А. laidlawii* GapN sequences (A9NHU4_ACHL; A9NHU7_ACHLI : LR103Y; S151Y; T180E; D215K; R437H); taken together with phosphoglycerate accomulation it indicates that such substitutions increase GapN activity.

Accumulation of thymidine and numerous substitutions in *A. laidlawii* thymidine kinase A9NEC0_ACHLI indicate that these substitutions decrease thymidine kinase activity.

Metabolite accumulation analysis revealed UMP and CMP nodes in *S. melliferum*, uridine and cytidine nodes in *A. laidlawii*, and absence of maximums in the *M. gallisepticum* pyrimidine module. Uridine kinase (Udk) catalyzes phosphorylation of uridine and cytidine with formation of mononucleotides UMP and CMP. Uridilate kinase (PyrH) catalyzes phosphorylation of UMP and CMP in cells and can be responsible for their accumulation. The metabolomic data indicate increased activity of the enzyme uridine kinase or reduced activity of uridilate kinase in *S. melliferum* cells. Since the mutations were detected in both enzymes (Udk: A16G substitution in Q7NB32_MYCGA and Y159F substitution in G9Y068_SPIME relative to *T. thermophilus*, PyrH: N137S substitution in A9NHC4_ACHLI and N137Q substitution in G9XZS5_SPIME relative to *B. subtilis*) responsible for the consumption and synthesis of UMP, it is difficult to accurately determine the nature of the mutations effect.

Analysis of accumulation of *S. melliferum* metabolites shows that cells have metabolites maximums in guanine and GMP, while in *A. laidlawii* and *M. gallisepticum* guanine accumulates to a greater degree than the GMP. **Hypoxanthine-guanine phosphoribosyltransferase (Hpt)** catalyzes reaction of transition of guanine to GMP. It can be assumed that Hpt has higher activity in *S. melliferum* than in *the A. laidlawii and M. gallisepticum*. We conclude that mutation I158F has an increasing effect on the *S. melliferum* Hpt activity, while mutation in the *A. laidlawii* Hpt sequence has a reducing activity.

In *M. gallisepticum* metabolome we identified adenine maximum that is a substrate for adenine phosphoribosyltransferase (Apt). We can assume that this mutation reduces the substrate binding activity of the Apt in *M. gallisepticum*. We identified pair maximum AMP=adenine in *S. melliferum*, which may indicate the increasing activity of the adenine phosphoribosyltransferase in this bacteria. Also we found two changes in the active sites *S. melliferum* adenine phosphoribosyltransferase: L27I and T131S. We assume that one of these substitutions may be associated with increased activity of the Apt in *S. melliferum*.

REFERENCES S1

1. Tchieu JH, Norris V, Edwards JS, Saier MH, Jr. (2001) The complete phosphotransferase system in Escherichia coli. J Mol Microbiol Biotechnol 3: 329-346.

2. Alexeev D, Kostrjukova E, Aliper A, Popenko A, Bazaleev N, et al. (2012) Application of Spiroplasma melliferum proteogenomic profiling for the discovery of virulence factors and pathogenicity mechanisms in host-associated spiroplasmas. J Proteome Res 11: 224-236.

3. Lazarev VN, Levitskii SA, Basovskii YI, Chukin MM, Akopian TA, et al. (2011) Complete genome and proteome of Acholeplasma laidlawii. J Bacteriol 193: 4943-4953.

4. Papazisi L, Gorton TS, Kutish G, Markham PF, Browning GF, et al. (2003) The complete genome sequence of the avian pathogen Mycoplasma gallisepticum strain R(low). Microbiology 149: 2307-2316.

5. Rottem S, Razin S (1969) Sugar transport in Mycoplasma gallisepticum. J Bacteriol 97: 787-792.

6. Kornberg HL, Lambourne LT (1992) Role of the phosphoenolpyruvate-dependent fructose phosphotransferase system in the utilization of mannose by Escherichia coli. Proc Biol Sci 250: 51-55.

7. Molin M, Norbeck J, Blomberg A (2003) Dihydroxyacetone kinases in Saccharomyces cerevisiae are involved in detoxification of dihydroxyacetone. J Biol Chem 278: 1415-1423.

8. Yus E, Maier T, Michalodimitrakis K, van Noort V, Yamada T, et al. (2009) Impact of genome reduction on bacterial metabolism and its regulation. Science 326: 1263-1268.

9. Iwasaki W, Miki K (2007) Crystal structure of the stationary phase survival protein SurE with metal ion and AMP. J Mol Biol 371: 123-136.

10. Falke D, Labenz J, Brauer D, Muller WE (1982) Adenosine diphosphate: thymidine 5'-phosphotransferase, a new enzyme activity, associated with the Herpes simplex virus-induced deoxypyrimidine kinase. Biochim Biophys Acta 708: 99-103.

**TABLES**

**Table S1. Suggested changes in enzymatic activity based on the compound concentrations in three Mollicutes species.**

|  | **Protein activity, conclusion** | | | **Nodes metabolite, in responsible organism** | | |
| --- | --- | --- | --- | --- | --- | --- |
| **Protein name** | **Acl** | **Spiro** | **Mga** | **Acl** | **Spiro** | **Mga** |
| **Purine nucleoside phosphorylase deoD** | + | - | - | Guanine | dGMP> dGuanosine | Guanosine, dGuanosine |
| **GMP synthase** | - | - | - | Glutamate,GMP | Glutamate,GMP | Glutamate,GMP |
| dITP/XTP pyrophosphatase,rdgB | - | + | - | XTP | - | ITP |
| Hypoxanthine-guanine phosphoribosyltransferase Hpt/ **Adenine phosphoribosyltransferase apt** | - | - | - | Guanine,Adenine | Guanine,Adenine | Guanine,Adenine |
| **1-phosphofructokinase** fruK | - | + | + | F-1-P |  |  |
| **Glycerol-3-phosphate acyltransferase plsY**/ **Phosphate acyltransferase plsX** | - | - | - | Glycerol-3-P | Glycerol-3-P | Glycerol-3-P |
| **Fructose-bisphosphate aldolase,fba** | + | - | - | - | G-3-P | G-3-P |
| **Fructose-bisphosphate aldolase,fba** |  |  |  | sedoheptulose-7-P |  |  |
| Ribose-phosphate pyrophosphokinase,prsA | - | + | + | Ribose-5-P | - | - |
| **Transketolase, tkt_2/**  Transketolase **tkt_1** | - | 0 | 0 | Fructose-6-p,S-7-p,Xylulose-5-P |  |  |
| Glyceraldehyde 3-P dehydrogenase/ Glyceraldehyde-3-phosphate dehydrogenase (NADP),putA | + | - | - | Glycerate-3-P(2-P) | Glyceraldehyde-3-P | Glyceraldehyde-3-P |
| **Enolase,eno** | - | + | + | Glycerate-3-P(2-P) | - | - |
| **Thymidine kinase,tdk** | - | + | + | Thymidine | - | - |
| **Uridine kinase,udk/** Uridylate kinase, PyrH | + | + | - | Uridine | UMP | - |
| **Uridine kinase,udk** | - | + | - | Cytidine | CMP | - |
