# Supplementary figures and images for: Metabolomic Analysis of Three Mollicute Species
Source: PLoS One. 2014 Mar 4;9(3):e89312. doi: 10.1371/journal.pone.0089312 (PMC3942410; doi:10.1371/journal.pone.0089312)

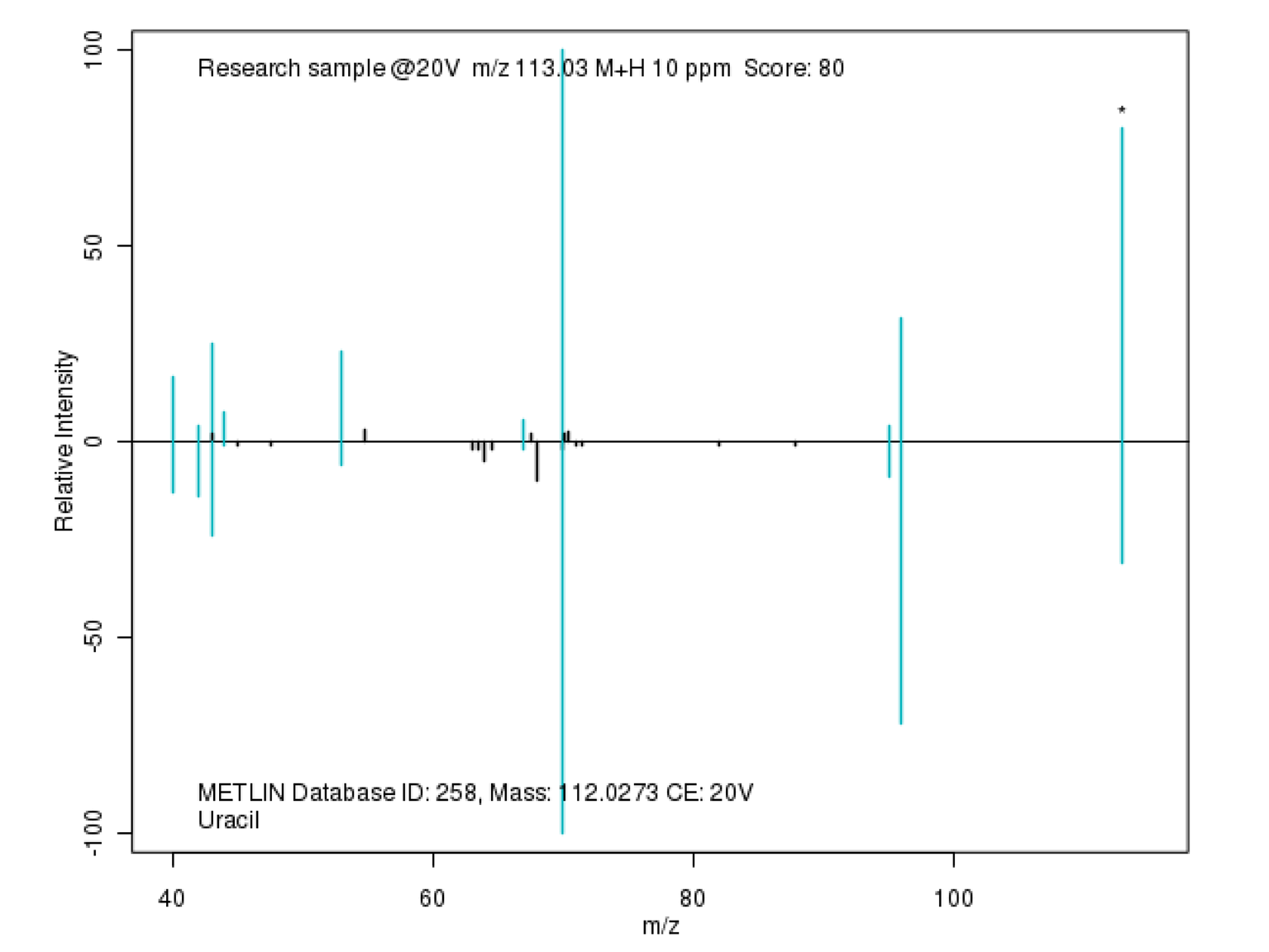

Supplement: Figure S1 — MS/MS Spectrum match of Uracil fragmentation spectrum obtained for the S. melliferum sample. Uracil fragmentation spectrum obtained for the S. melliferum sample is above the OX axis and standard Uracil fragmentation spectrum in positive ionization mode collision energy 20 eV from Metlin Metabolites database is under the OX axis [30]; Uracil M+H: m/z = 113.03344, Δ = 10 ppm. (TIF) [file pone.0089312.s002.tif]

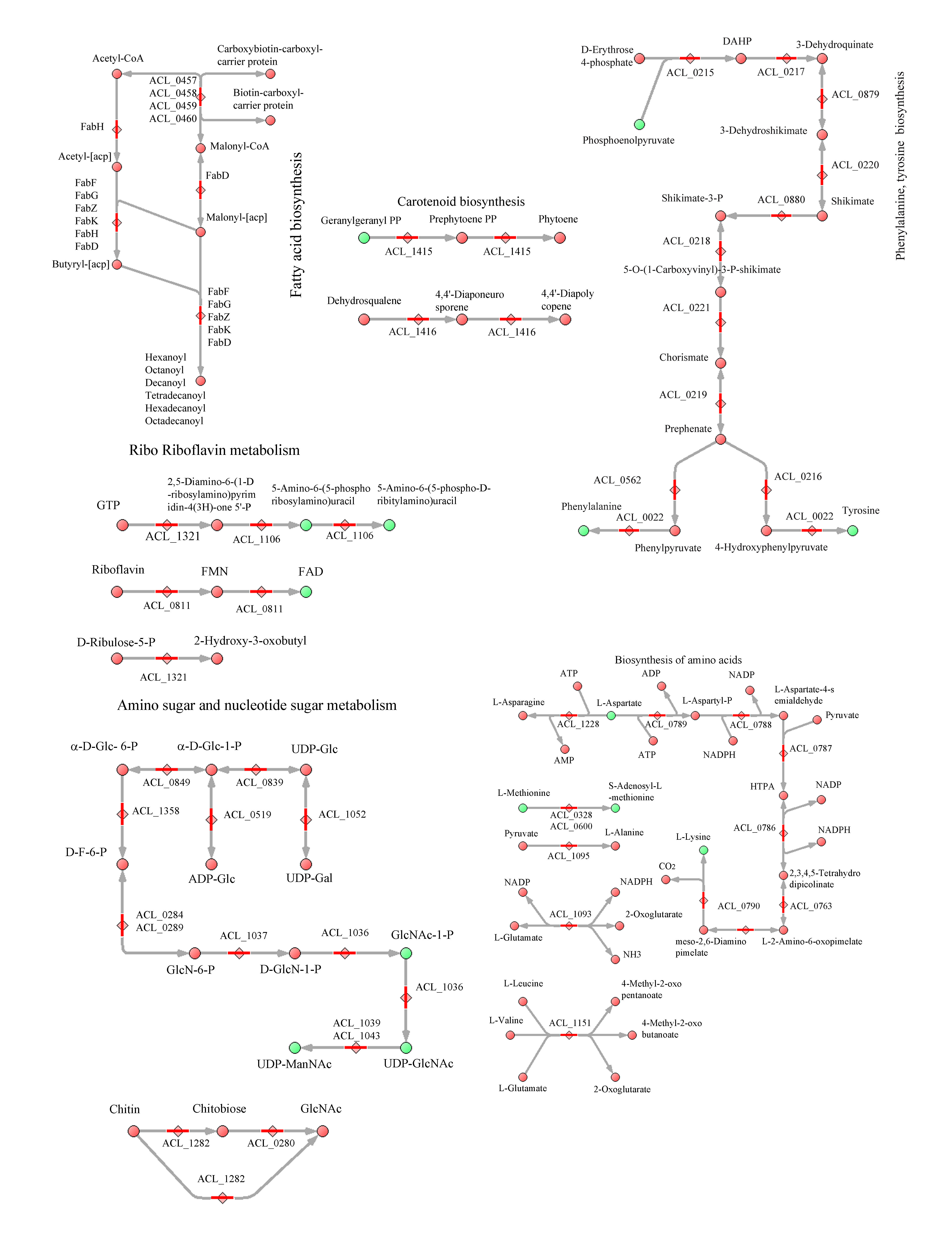

Supplement: Figure S2 — Reconstructed pathways characteristic for A. laidlawii which are absent in M. gallisepticum . For description see legend to Figure 1. (TIF) [file pone.0089312.s003.tif]

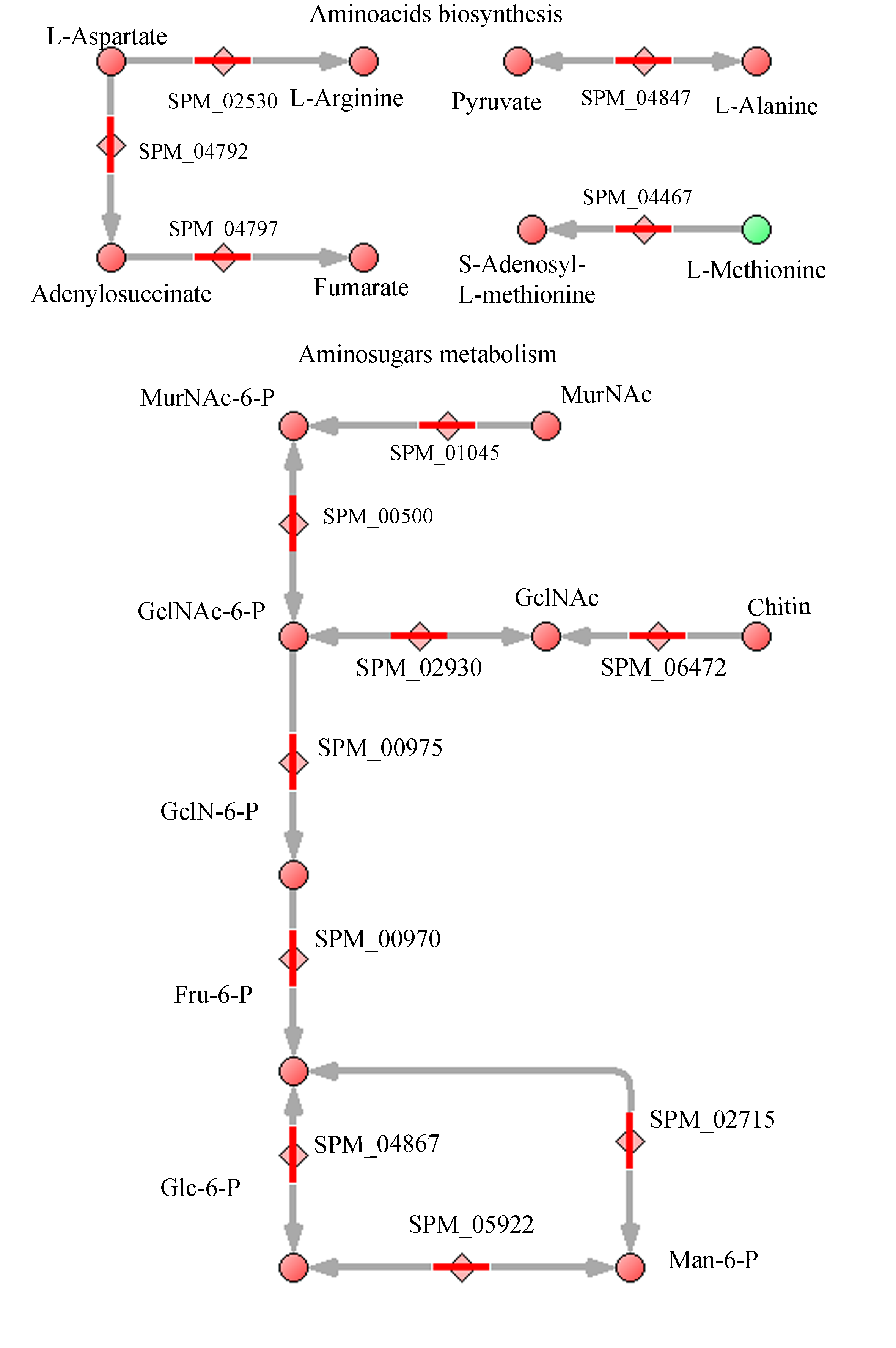

Supplement: Figure S3 — Reconstructed pathways characteristic for S. melliferum which are absent in M. gallisepticum . For description see legend to Figure 1. (TIF) [file pone.0089312.s004.tif]
